# Robot-assisted laparoscopy does not have demonstrable advantages over conventional laparoscopy in endometriosis surgery: a systematic review and meta-analysis

**DOI:** 10.1007/s00464-023-10587-9

**Published:** 2023-12-07

**Authors:** Ádám Csirzó, Dénes Péter Kovács, Anett Szabó, Péter Fehérvári, Árpád Jankó, Péter Hegyi, Péter Nyirády, Zoltán Sipos, Levente Sára, Nándor Ács, István Szabó, Sándor Valent

**Affiliations:** 1 Centre for Translational Medicine, Semmelweis University, Üllői út 26, 1082 Budapest, Hungary; 2 Department of Obstetrics and Gynecology, Semmelweis University, Budapest, Hungary; 3 Institute of Pancreatic Diseases, Semmelweis University, Budapest, Hungary; 4 Department of Urology, Semmelweis University, Budapest, Hungary; 5 Medical School, Institute for Translational Medicine, University of Pécs, Pecs, Hungary; 6 Department of Biostatistics, University of Veterinary Medicine Budapest, Budapest, Hungary; 7 Medical School, Institute of Bioanalysis, University of Pécs, Pecs, Hungary

**Keywords:** Deep infiltrating endometriosis, DIE, Single-port, Multiport, rASRM, Da Vinci Surgical System

## Abstract

**Background:**

Endometriosis is a chronic condition affecting 6–10% of women of reproductive age, with endometriosis-related pain and infertility being the leading symptoms. Currently, the gold standard treatment approach to surgery is conventional laparoscopy (CL); however, the increasing availability of robot-assisted surgery is projected as a competitor of CL. This study aimed to compare the perioperative outcomes of robot-assisted laparoscopy (RAL) and CL in endometriosis surgery.

**Objectives:**

We aimed to compare the effectiveness and safety of these two procedures.

**Methods:**

A systematic search was conducted in three medical databases. Studies investigating different perioperative outcomes of endometriosis-related surgeries were included. Results are presented as odds ratios (OR) or mean differences (MD) with 95% confidence intervals (CI).

**Results:**

Our search yielded 2,014 records, of which 13 were eligible for data extraction. No significant differences were detected between the CL and RAL groups in terms of intraoperative complications (OR = 1.07, CI 0.43–2.63), postoperative complications (OR = 1.3, CI 0.73–2.32), number of conversions to open surgery (OR = 1.34, CI 0.76–2.37), length of hospital stays (MD = 0.12, CI 0.33–0.57), blood loss (MD = 16.73, CI 4.18–37.63) or number of rehospitalizations (OR = 0.95, CI 0.13–6.75). In terms of operative times (MD = 28.09 min, CI 11.59–44.59) and operating room times (MD = 51.39 min, CI 15.07–87.72;), the RAL technique remained inferior.

**Conclusion:**

RAL does not have statistically demonstrable advantages over CL in terms of perioperative outcomes for endometriosis-related surgery.

**Graphical abstract:**

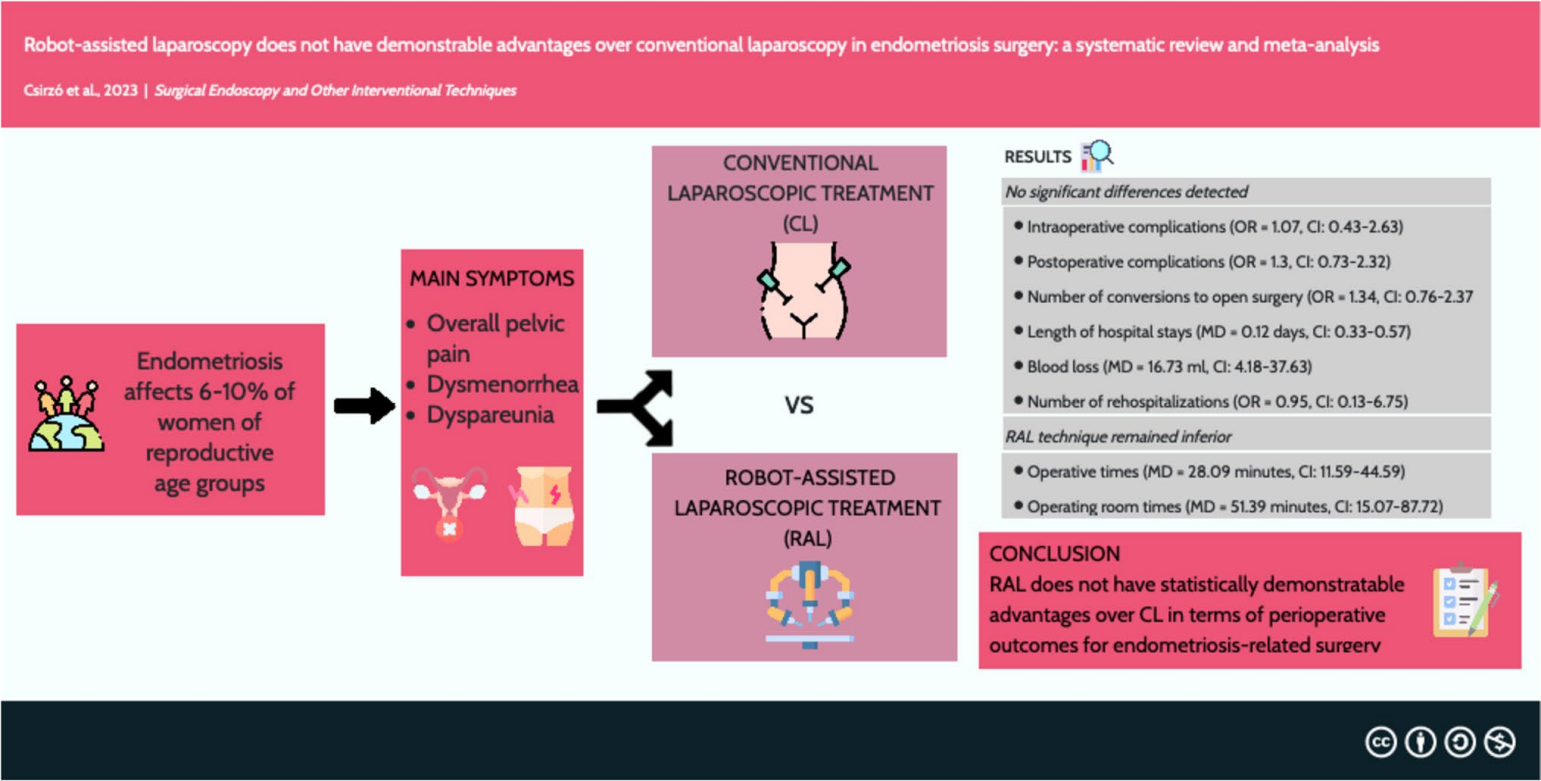

**Supplementary Information:**

The online version contains supplementary material available at 10.1007/s00464-023-10587-9.

Endometriosis is an estrogen-dependent benign gynecological disorder associated with pelvic pain and infertility. Globally, approximately 70 million women of reproductive age suffer from various forms of endometriosis [1]. It is characterized by the presence of functioning endometrium-like tissue outside the uterine cavity, which induces an inflammatory response [2]. The leading complaints of endometriosis are dysmenorrhea, dyspareunia, dysuria, and dyschezia, accompanied by infertility. Several therapeutic options, including medications, surgical interventions, and non-medical management strategies, aim to reduce pain-related symptoms and restore fertility [3]. Surgical approaches consist mainly of minimally invasive techniques. Their advantages include lower rates of complications, such as shorter hospital stays, reduced trauma, and lower chances of infections compared to open surgeries [4]. Conventional laparoscopic surgery is considered as the standard of care for the above reasons; however, its limitations include 2-dimensional (2D) visualization, ergonomic challenges for the surgeon, and limited instrument range [5]. As more advanced techniques such as robot-assisted laparoscopy (RAL) are becoming more and more prevalent, the above limitations of laparoscopy are expected to be overcome.

RAL has the advantages of minimally invasive surgery, but also has other benefits. On the surgical side, robot-assisted surgery, using 3D technology, offers better visualization of the surgical site, instrumentation facilitates seven degrees of freedom, permits tremor-free handling, and reduces work fatigue while also having a shortened learning curve compared to laparoscopic surgery [4]. Previous studies have shown that RAL has clinically relevant advantages in numerous other surgical areas (e.g., rectal cancer resection and distal pancreatectomy) [5, 6]. Advantages reported in the outcome of robot-assisted operations compared to CL include reduced postoperative pain and blood loss [7]. However, the two main disadvantages of the robot include the absence of tactile feedback and the high cost of installing and maintaining machinery.

Although the benefits of RAL have been demonstrated in several surgical fields, its benefits over CL in endometriosis have not yet been investigated. Therefore, we aimed to compare the effectiveness and safety of these two procedures.

## Materials and methods

Our systematic review and meta-analysis was reported according to the PRISMA (Preferred Reporting Items for Systematic Reviews and Meta-Analyses) 2020 Statement. (Supplementary Table 1.) This study followed the recommendations of the Cochrane Handbook for Systematic Reviews of Interventions, Version 6.3 [8]. The review protocol was registered on PROSPERO (York, UK) under the registration number CRD42023397045.

### Literature search and eligibility criteria

A systematic literature search was performed using three medical databases, MEDLINE (via PubMed), Cochrane Library (CENTRAL), and Embase on February 15, 2023. The main domains of the search key were endometriosis, robot-assisted surgery, and laparoscopy. The full search key is presented in Supplementary Table S5. Case reports, case series, conference abstracts, trial protocols, letters, and reviews were excluded. No language or other restrictions were imposed.

Papers were eligible if they conformed to our PICO (Population, Intervention, Comparison, Outcome) framework. Articles on premenopausal women who underwent surgery for endometriosis (P) were included. The diagnosis of endometriosis was based on either of the following: clinical symptoms, imaging techniques, laparoscopic findings, or histology. The included studies required robot-assisted surgery as an intervention (I) compared to the conventional laparoscopic approach (C). Our outcomes were different perioperative outcomes: intra-, and postoperative complications, operating room time, operative time, anesthesia time, number of recurrences, estimated blood loss, and length of hospital stay following surgery (O). An important criterion was that the articles had to define the outcomes mentioned above in the same way for the two surgical approaches. Detailed exclusion and inclusion criteria are presented in Supplementary Table S2.

### Study selection and data collection

EndNote X9 (Clarivate Analytics, Philadelphia, PA, USA) was used for duplicate removal, rayyan.ai for title-abstract selection, and EndNote X9 for full-text selection. At every level of selection, two independent authors (ÁC, DPK) screened the publications, and disagreements were resolved by a third author (ÁJ).

Two authors (ÁC, DPK) independently extracted data into a predefined Excel spreadsheet (Office 365, Microsoft, Redmond, WA, USA). The following data were extracted from each eligible article: first author, year of publication, study type, study location, number of centers involved, study design, demographic data (sample size, age, body mass index (BMI), presence of infertility, previous surgeries, details of procedures, and number of surgeons performing the operations) and data for the outcomes for statistical analysis. A third reviewer (ÁJ) resolved the discrepancies. Cohen's kappa coefficient (κ) was calculated after each step to measure interrater reliability [9].

### Quality assessment and quality of evidence

The quality of the outcomes was assessed separately by two reviewers (ÁC, ÁJ) using the risk of bias tool Risk Of Bias In Non-randomized Studies—of Interventions (ROBINS-I) for non-randomized- and RoB 2 for randomized trials. Any disagreements were resolved by a third reviewer (DPK). The VISualization (Robvis) tool was used to visualize the results [10].

The recommendations of the "Grades of Recommendation, Assessment, Development, and Evaluation (GRADE)" workgroup were followed to evaluate the quality of evidence [11].

### Data synthesis and analysis

The odds ratio with 95% CI was used to measure the effect of intra- and postoperative complications, whereas mean differences (MDs) were used for outcomes measuring operation durations. To calculate the odds ratio, the total number of patients in each group and those with the event of interest were extracted from each study, whereas we used the between-group mean differences and SDs to calculate the effect measure for continuous outcomes. Raw data from the selected studies were pooled using a random-effects model with the Mantel–Haenszel method and the Hartung–Knapp adjustment [12, 13]. To estimate *τ*^2^, we used the Paule-Mandel method and the Q profile method to calculate the confidence interval of *τ*^2^. A funnel plot of the logarithm of effect size and comparison with the standard error for each trial was used to evaluate publication bias. Statistical heterogeneity across trials was assessed by means of the Cochrane Q test, and the *I*^2^ statistic values. Outlier and influence analyses were carried out following the recommendations of Harrer et al. and Viechtbauer and Cheung [13, 14]. Forest plots were used to graphically summarize results. Where applicable, we reported the prediction intervals (i.e., the expected range of effects of future studies) of results as recommended by IntHout et al. 2016 [15]. All analyses were carried out using R 4.1.3, the packages ‘meta’ and ‘dmetar’ [16–18]. 

## Results

### Search and selection

**Fig. 1:**
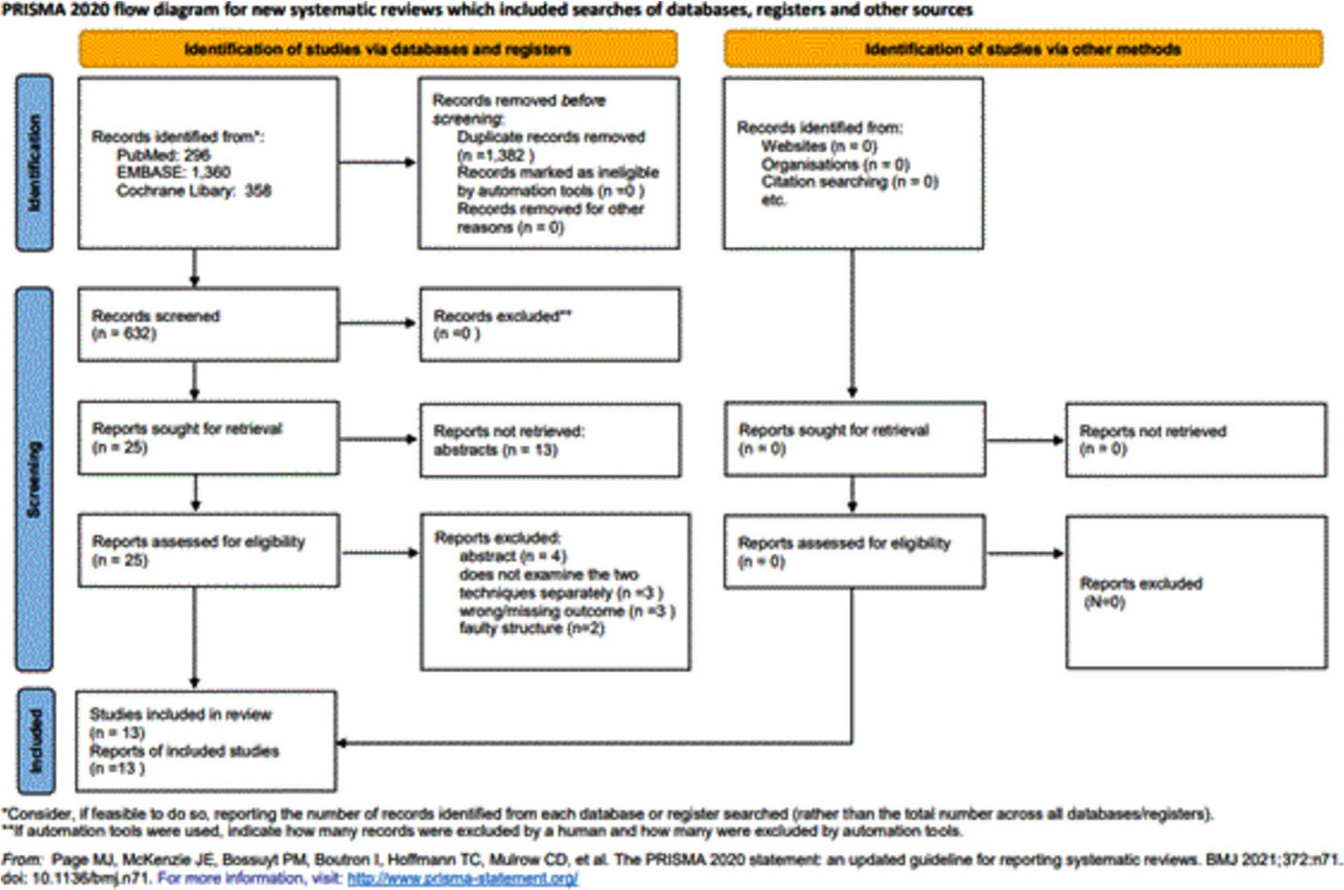
PRISMA flowchart of the selection process

A total of 2,014 studies were identified. After removing 1,382 duplicates, we found 632 eligible studies by title abstract, of which 38 were eligible for full-text selection. Finally, the data from 13 articles were extracted (Fig. 1).

### Basic characteristics

Of these articles, one was a randomized controlled trial (RCT), four were prospective, and eight were retrospective cohort studies, published between 2010 and 2022. Most of them implemented multiport laparoscopic surgeries, with two exceptions, which applied the single port technique [19, 20]. Ten articles contained information on the type of robot used, which was the da Vinci type in all cases, and also, ten studies reported on the number of surgeons performing the surgeries, ranging from one to five. Ten articles also evaluated the experience of the surgeons as the expertise of the surgeons based on subjective reports or metric scales, but all surgeons were experts.

The CL group included 1,009 patients and the RAL group 1,012. The baseline data are summarized in Table 1. The mean ages and BMIs of the two groups were similar, as well as the severity of endometriosis operated on. However, the latter was different between articles, five articles included only severe cases of endometriosis, but this did not provide a basis for selection [21–25]. In terms of study designs, the study by Soto et al*.,* the only RCT, represented the highest quality, although its primary limitation was the inclusion of suspected endometriosis cases as well [26]. Data on anesthesia time and postoperative recurrence were not available for analysis. Furthermore, the examined articles did not provide data on the impact of interventions on the quality of life.

**Table 1:** Baseline characteristics of included studies

Author	Year	Study type	rASRM stage	No. of. surgeons	Group	Sample size	Age	BMI
Ferrier [43]	2022	p	II./III./IV	3	RAL	61	36 ± 7	25 ± 5
					CL	61	35 ± 7	26 ± 8
Raimondo [21]	2021	p	III./IV	1	RAL	22	38 ± 7	24.5 (21;27)
					CL	22	36 ± 5	22.5 (21;24)
Hiltunen [29]	2021	r	I./II./III./IV	N.A	RAL	18	N.A	24 [18;38]
					CL	76	N.A	26 [19;39]
Lee [20]	2020	p	N.A. (just ovarian)	N.A	SP-RAL	40	28.6 ± 5.8	21.28 ± 3.78
					SP-CL	54	30.69 ± 5.82	20.37 ± 2.36
Le Gac [22]	2020	p	III./IV	2	RAL	23	25 ± 3	25 ± 3
					CL	25	37 ± 8	25 ± 4
Moon [19]	2018	r	I./II./III./IV	3	SP-RAL	68	32.4 ± 6.8	23.1 ± 3.4
					SP-CL	52	33.1 ± 7.9	21.1 ± 3
Soto [26]	2017	RCT	I./II./III./IV	5	RAL	35	34.3 ± 7.2	26.1 ± 5.2
					CL	38	34.5 ± 8.5	24.8 ± 5.9
Carpentier [32]	2016	r	N.A. (just bladder)	1	RAL	15	28.5 [N.A.;N.A.]	23.8 (N.A.;N.A.)
					CL	22	29 [N.A.;N.A.]	21.9 (N.A.;N.A.)
Nezhat [23]	2015	r	III./IV	1	RAL	147	30 [21;38]	23 [19;32]
					CL	273	31 [19;42]	23 [19;29]
Magrina [24]	2015	r	III./IV	3	RAL	331	40 ± 10.1	26.1 ± 5.9
					CL	162	38.3 ± 10.7	25.5 ± 5.7
Nezhat [25]	2014	r	III./IV	1	RAL	147	39 [34;44]	27.36 [23.9;34.09]
					CL	86	38 [31;44]	24.53 [22.27;26.96]
Dulemba [44]	2013	r	I./II./III./IV	1	RAL	180	32.6 ± 9.7	27.9 ± 7.7
					CL	100	29.2 ± 9.2	26.8 ± 11.9
Nezhat [45]	2010	r	I./II./III./IV	N.A	RAL	40	35	24 [19;37]
					CL	38	33	23 [18;31]

Fig. explanation: 0 ± 0, means mean ± SD, 0 [0;0], means median [range min.; range max.], 0 (0;0), means median (interquartile min.;interquartile max.). ‘p’ means prospective cohort study, ‘r’ means retrospective cohort study and RCT means randomized controlled trial. SP-RAL means single-port robot-assisted laparoscopy. SP-CL means single-port conventional laparoscopy. ‘N.A.’ means no available data

### Complications

**Fig. 2:**
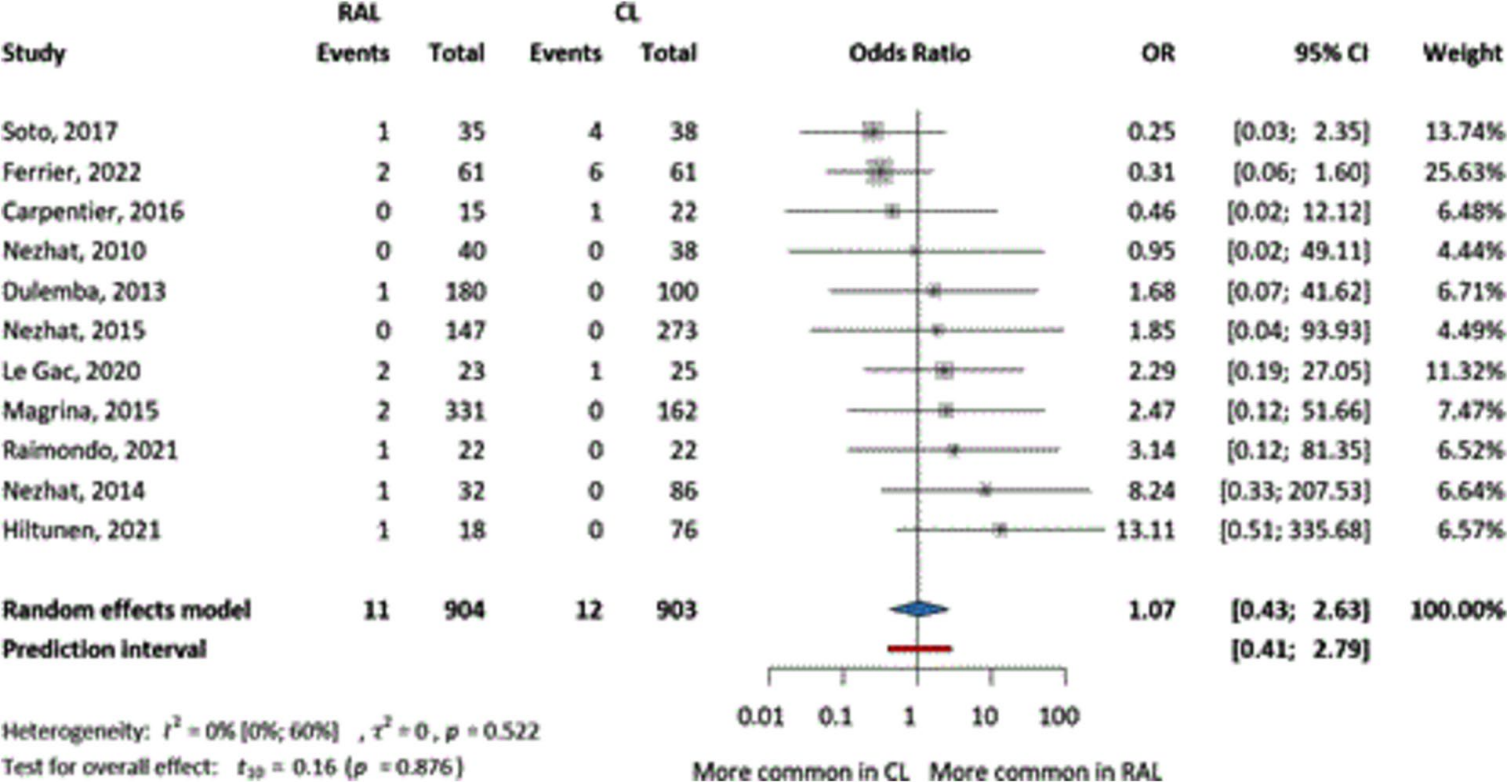
The comparison of RAL and CL in terms of odds ratio of intraoperative complications (event numbers)

First, we examined intraoperative complications, which were evaluated in 11 articles, without specifying the type of complications, only their number. The two groups showed no difference (OR = 1.07, CI 0.43–2.63) in the number of complications (Fig. 2). The relative frequency of complications in the RAL group was 1.21% and 1.32% in the CL group.

**Fig. 3:**
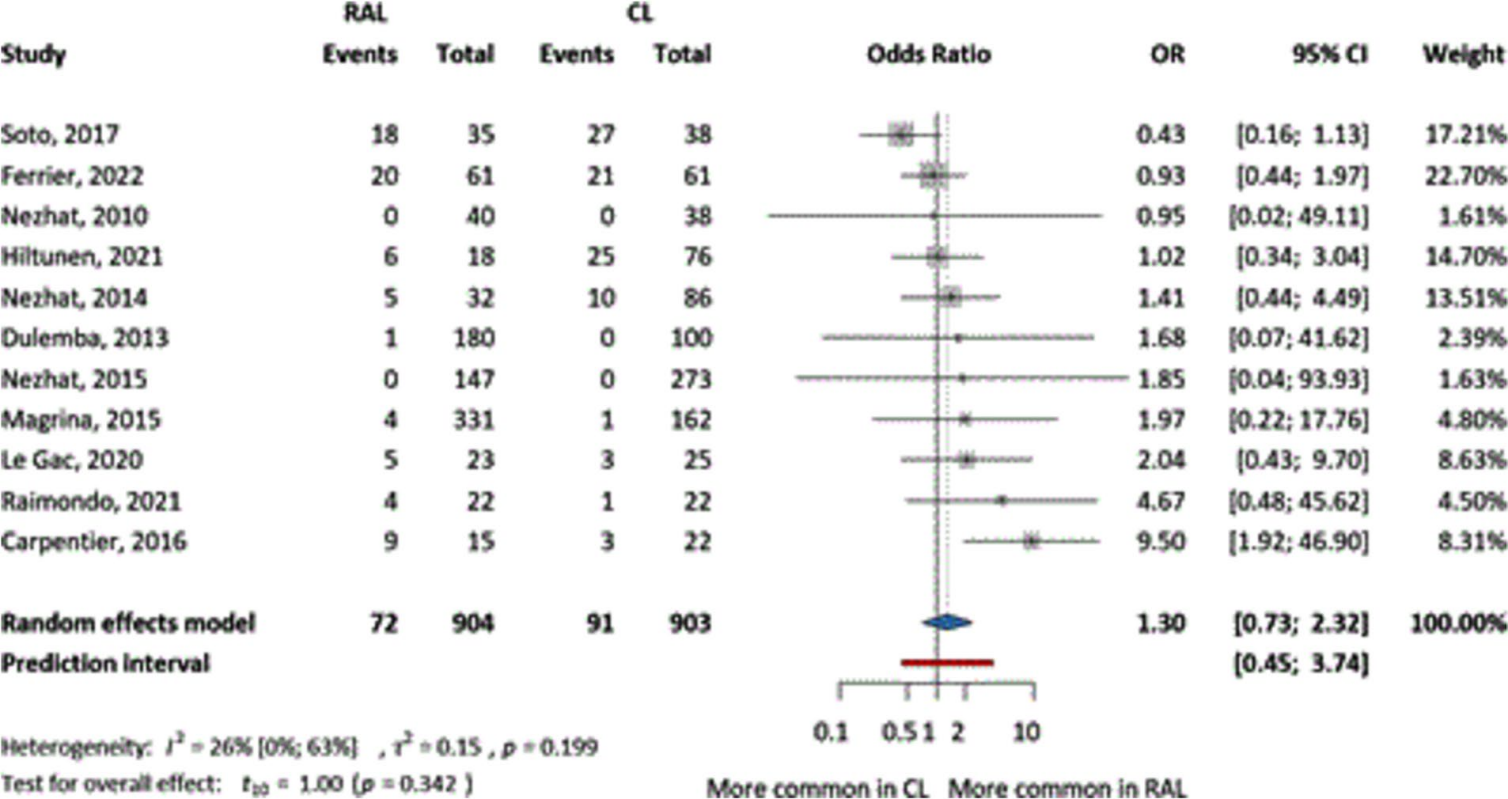
The comparison of RAL and CL in terms of odds ratio of postoperative complications (event numbers)

Postoperative complications were also investigated in 11 articles without specifying the exact timing or type of complications, only their number. No differences in postoperative complications were detected between the CL and RAL groups (OR = 1.3, CI 0.73–2.32) (Fig. 3). The relative frequency of postoperative complications in the RAL group was 7.96%, and 10.07% in the CL group. Furthermore, four articles categorized these complications based on the Clavien-Dindo classification, with results similar to those of previous studies (Supplementary Figures S10-12).

Ten articles investigated the number of laparotomy conversions to open surgery. Neither CL nor RAL had clinically relevant, higher conversion rates (OR = 1.34, CI 0.76–2.37) (Supplementary Fig. S13). The relative frequencies of conversions in the RAL group were 0.74% and 0.49% in the CL group, respectively.

Three articles evaluated the number of rehospitalizations. No significant difference was observed between the two procedures (OR = 0.95, CI 0.13–6.75) (Supplementary Figure S14).

### Estimated blood loss

Eleven articles examined the estimated blood loss in milliliters during surgery. One article (*Lee 2020*) reported blood loss in grams of hemoglobin per deciliter; therefore, these data were omitted from the analysis. Approximately 16 ml more blood was lost during RAL surgeries (MD = 16.73, CI 4.18–37.63) (Supplementary Fig. S15) However, this finding was neither clinically relevant nor statistically significant.

### Length of the procedures

**Fig. 4:**
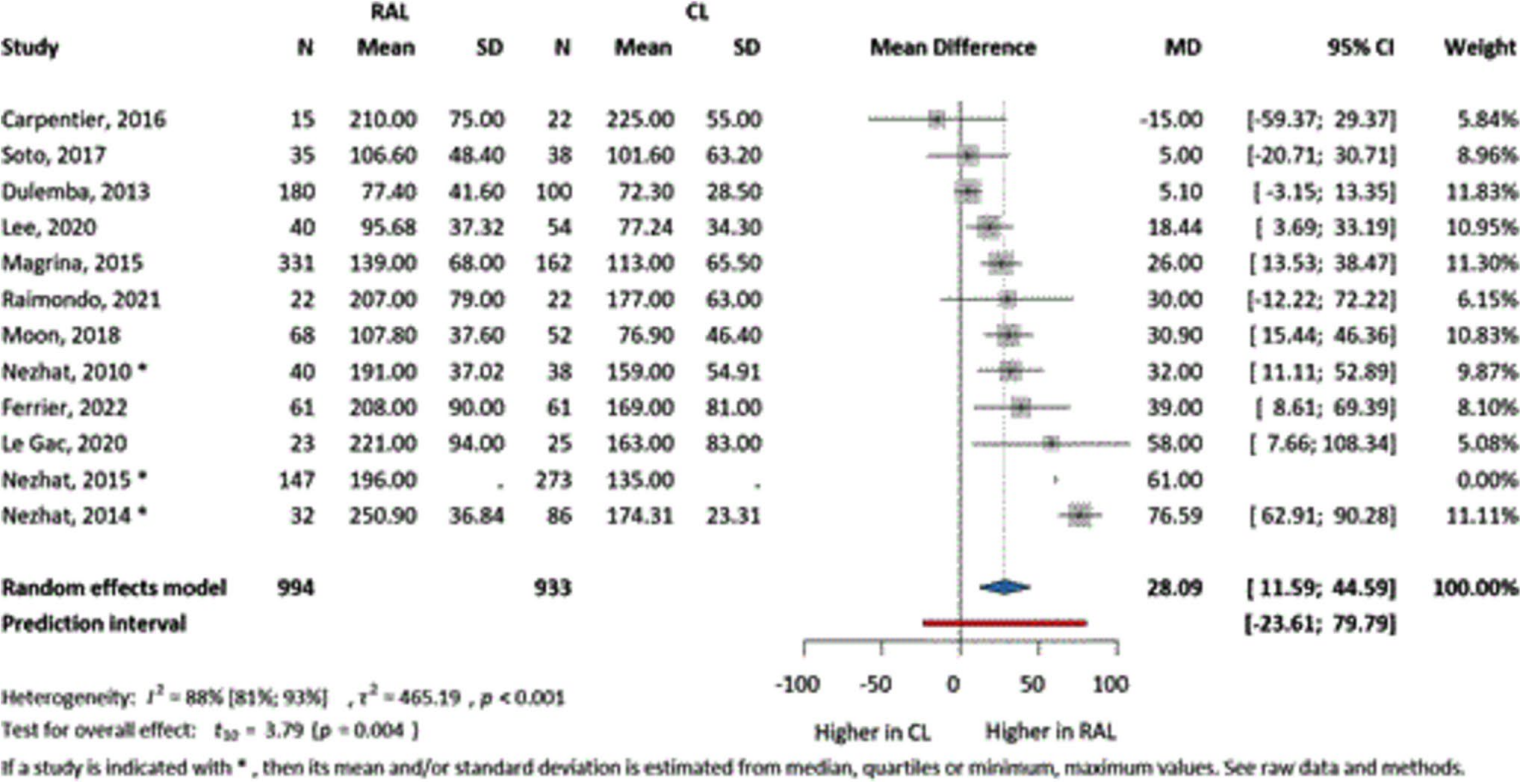
The comparison of RAL and CL in terms of mean difference of operating times (minutes)

Twelve articles evaluated the operative time, measured in minutes from skin incision to wound closure. For the robot-assisted technique, time included the times for both docking and undocking. The operative times showed that the robot-assisted technique took almost half an hour (MD = 28.09, CI 11.59–44.59) (Fig. 4) longer compared to CL. This result was clinically relevant and statistically significant.

**Fig. 5:**
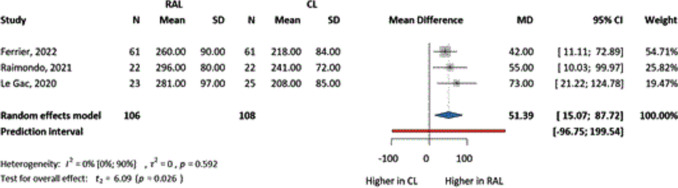
The comparison of RAL and CL in terms of mean difference of operating room times (minutes)

Three studies examined the time spent in the operating room, measured in minutes, from patient arrival to departure from the operating room. Docking and undocking times were also included for RAL. Similarly, these results favored CL (MD = 51.39, CI 15.07–87.72) (Fig. 5), being clinically relevant and statistically significant.

### Length of hospital stay

Eight studies analyzed the number of days spent in hospital following surgery (MD = 0.12, CI 0.33–0.57) (Supplementary Fig. S16), showing neither clinically relevant nor statistically significant differences.

### Quality and risk of bias assessment

The risk of bias was assessed using the ROBINS-I tool for observational studies. Most articles were rated as "moderate." Three articles lacked information on how patients were assigned to the RAL or CL groups; thus, two received a "serious" risk for bias classification, and one a "critical" rating because information on the number of physicians performing the operations was missing. One additional article received a "serious" rating because it did not include information on the number of surgeons involved. (Supplementary Figs. S1-8).

For the RCT, the risk of bias was assessed using the RoB 2 tool, resulting in a low-risk rating. (Supplementary Fig. S9).

GRADEpro was used for quality control with moderate rating results for all evaluated outcomes. (Supplementary Table S3).

Intra- and postoperative complications, the number of rehospitalizations, and the number of conversions had a low heterogeneity. However, heterogeneity was high for operative times, estimated blood loss, and length of hospital stay. The high heterogeneity could be due to the differences in the severity of endometriosis. Unlike for operative time, low heterogeneity was observed for operating room time, probably due to the inclusion of endometriosis with the same severity.

## Discussion

This systematic review and meta-analysis identified 13 studies that compared RAL with CL in terms of perioperative outcomes of endometriosis surgery. The quantitative synthesis of our findings confirmed that RAL had no numerical advantages over CL in the aspects studied. Moreover, we found it to be inferior in terms of operating room and operative times. Subgroup analysis based on the pattern of endometriosis was not feasible with the available data.

In addition to previous studies, we examined operating room time as a new outcome [27–29]. However, we obtained similar results for all other perioperative outcomes. Our results did not show the expected benefit of RAL over CL in terms of intra- and postoperative complications, estimated blood loss, number of rehospitalizations, or days spent in hospital, and we observed even longer operative times by approximately 30 min. The latter can be attributed to an average docking time of approximately 18.2 min [30]. Operating time was found to be the most significant factor associated with postoperative complications and length of postoperative hospital stay. Magrina et al. found that for every additional 60 min of operating time, the odds of postoperative complications and prolonged hospital stay increased the chances by 57% and 103%, respectively [24]. This is partly explained by the disproportionate distribution of patients. In some articles, more radical procedures (e.g., endometriosis surgery with concomitant hysterectomy) were performed and patients with more advanced endometriosis were operated on with RAL according to the revised American Society for Reproductive Medicine (rASRM) staging. This might indicate that the surgeon favored a robotic approach and might have added bias to the results, contributing to differences in operating times [24, 31]. However, we did not find significant differences in rASM classification between the two approaches. (Supplementary Figures S17-S20) On the other hand, the experience of Nezhat suggests that procedures for the treatment of severe disease require multiple camera and instrument exchanges, making CL easier to perform [23].

It should be noted that the only RCT conducted by *Soto 2017* found the mean operative time and blood loss within the range of time and volumes previously reported by other non-randomized studies. This suggests that their findings are unlikely to be related to patient selection and the experience of the surgeons or the team with various platforms [32]. Not surprisingly, in the articles describing more severe cases (e.g., bowel (deep infiltrating endometriosis (DIE), rASRM stage III/IV.), an even longer operative times could be observed compared to the mean difference we reported. Similar to the operative time, we obtained a significant difference of approximately 50 min in the operating room time. This difference could also be attributed to the necessary preparation procedures of the robot in addition to factors described influencing the length of the surgery time.

Intraoperative complications were crucial in determining intra- and postoperative outcomes, such as operative time (thus, operating room time), expected blood loss, the likelihood of conversion to open surgery, days spent in hospital, and postoperative complications. Most studies showed relatively low numbers of intraoperative and postoperative complications, indicating that both methods were safe and neither seemed to be superior in terms of complication rates. It should be noted that Carpentier et al. only operated on bladder DIE, and the relative frequency of postoperative complications in the RAL group was 60% versus 36% in the CL group [32]. Conversion to open surgery depended on several factors, including the previous abdominal surgeries of the patient and unexpected technical events, among other factors. However, the level of experience of the surgeon was a factor that needs to be highlighted.

Other meta-analyses had also been conducted on this topic; however, due to the low number of cases and methodological problems, we considered it necessary to conduct another meta-analysis. In the meta‐analysis by *Chen (2016)*, RAL was compared with CL for endometriosis surgery; no difference was found in most aspects, except for operating time [27]. In 2020, Restainato et al. and in 2018, Balla et al. performed a meta-analysis and found no difference in the operating time or complication rates between robot-assisted and conventional laparoscopy. However, in the latter study, in which all patients underwent colorectal resection due to endometriosis, only a small fraction (1.7%) of the procedures were performed with RAL. Moreover, complications were not evaluated separately for RAL and CL [28, 29].

Our study showed that RAL did not offer a quantifiable advantage in the day-to-day surgical management of patients with endometriosis. However, the reality is more nuanced; an important finding is that longer operative time has been correlated with increased overall costs strongly associated with the robotic platform [23]. As for costs, no data were found for endometriosis surgeries; however, such data were reported in closely related areas. A database study of 36,188 patients showed that robotic hysterectomy was more expensive than laparoscopic hysterectomy ($9,640 vs. $6,973, *P* < 0.01). In gynecological oncology, for endometrial or cervical cancer, the extra cost of using RAL was €1,456 per intervention [33].

Le Gac et al. mentioned earlier that the learning curve of robotic surgery in general could have influenced docking and operative times, as well as the complication rate [22]. The articles by Lee et al. and Terzi et al. demonstrated that the learning curves of RAL and CL differed significantly. For RAL surgeries, the operation time for hysterectomy could be reduced after 23 surgeries of the same type, whereas for CL, 75 surgeries were required [34, 35].

However, experts of both RAL and CL have experienced the convenience of using RAL, as it provides comfort and increased precision in the operating technique. The RAL offers better visualization of the surgical site using 3D technology and 15 times magnification. Also, from an ergonomic point of view, instruments that mimic the movement of human hands, wrists, and fingers allow an extensive range of motion that is more precise than natural hand and wrist movements. Owing to the robotic arms, the sustained maintenance of positions demanding substantial force does not precipitate deleterious consequences or substantial fatigue. CL also has indisputable advantages, such as haptic tissue feedback, which, for example, is particularly beneficial for establishing the pathological-healthy border during the excision of a DIE nodule. Also, the esthetic effect is highlighted mainly from the patients' point of view. For CL, both the location of the ports on the trunk and the smaller diameter of the trocars are options preferred by patients. Robot-assisted surgery appears to have fewer negative cognitive and musculoskeletal impacts on surgeons than CL [36]. In 2021, Sers et al. found that performing laparoscopic surgery on patients, especially with high BMIs, increased the prevalence of non-neutral postures and could have further increased the risk of musculoskeletal disorders in surgeons [37]. However, to date, no studies have investigated the more serious, long-term, irreversible effects of CL on health, such as the potential development of knee and hip joint impairment. Current recommendations for the use of RAL in the surgical treatment of endometriosis vary depending on several factors, including the individual circumstances of the patients, the expertise of the surgeon, and the availability of resources and equipment. In 2013, the American Association of Gynecologic Laparoscopists (AAGL) recommended that RAL should not replace CL or vaginal procedures for women who could otherwise undergo CL or vaginal surgery for benign gynecologic diseases [38]. On the basis of the guidelines of the Danish Health Authority, RAL hysterectomy should only be preferred over CL hysterectomy after careful consideration because its beneficial effect is uncertain due to longer operating time [39]. Especially, with regard to advanced stage endometriosis, RAL is a possible first-line approach for the surgical treatment of bowel DIE [23, 40]. Furthermore, Lee et al. conclude that robot-assisted cystectomy in bilateral ovarian endometrioma is better than the laparoscopic approach for preserving ovarian function [20]. The decision to use robot-assisted laparoscopy for the treatment of endometriosis should be made on a case-by-case basis, taking into account the specific needs and circumstances of the patient as well as the experience and skill of the surgeon. Patients are advised to consult their healthcare providers to determine the most appropriate treatment approach for their individual situations. It is essential to highlight that this was only a snapshot. As time passes, expert surgeons who have spent most of their lives with laparoscopy will spend more time with the robot-assisted technique and could produce entirely new results.

### Strengths and limitation

We followed our rigorous protocol, which had been registered in advance. Our investigation covered a long study period, with a high number of cases. Although there had been previous meta-analyses on the topic, we were able to include more articles than the latest one from 2020. Compared with previous meta-analyses, our review examined operating room time as a new outcome.

As for the limitations of our analysis, most articles were retrospective studies, and only one RCT was included. In most of the included articles, patient selection was based on the availability of a robotic room. Also, some articles performed only certain organ-specific interventions and operated only on a specific severity of endometriosis, thus not representing the full range. Furthermore, it is imperative to underscore that the same author has contributed to some of the selected articles.

## Conclusion

On the basis of our study, in most aspects, RAL seems to be equivalent to CL; however, in terms of time efficiency, it is inferior in the treatment of endometriosis.

### Implications for practice and research

Translating scientific knowledge for the benefit of patients has crucial importance [41, 42]. Our research suggests that in general practice, CL should be the first choice in the surgical treatment of patients with endometriosis. It is also financially advantageous, but is generally not considered in studies and has yet to be explored, especially when weighed against the cost of training a new surgeon and the lengthy learning curve compared to RAL.

However, RAL has practical advantages over CL, which has been poorly studied. From a surgical point of view, it has advantages in terms of posture, resulting in fewer orthopedics-related problems, and from the point of view of the patient, the higher magnification and better maneuverability may lead to more precise treatment. Further studies are needed to explore the medico-economic aspects of these two interventions.

### Supplementary Information

Below is the link to the electronic supplementary material.

Supplementary file1 (DOCX 4129 KB)

## Data Availability

The datasets used in this study can be found in the full-text articles included in the systematic review and meta-analysis.
