# Supplementary material for: Robot-assisted laparoscopy does not have demonstrable advantages over conventional laparoscopy in endometriosis surgery: a systematic review and meta-analysis
Source: Surg Endosc. 2023 Dec 7;38(2):529–39. doi: 10.1007/s00464-023-10587-9 (PMC10830624; doi:10.1007/s00464-023-10587-9)

**Supplementary Material**

**Title**

Robot-assisted Laparoscopy does not have demonstrable advantages over Conventional Laparoscopy in endometriosis surgery: a systematic review and meta-analysis

**Authors**

Ádám Csirzó^1,2^, Dénes Péter Kovács^1,2^, Anett Szabó^1,5^, Péter Fehérvári^1,7^, Árpád Jankó^1^, Péter Hegyi^1,4^, Péter Nyirády^1,5^, Zoltán Sipos^6,8^, Levente Sára^1,2^, Nándor Ács^1,2^, István Szabó^2^, Sándor Valent^1, 2^

**Affiliations:**

1. Centre for Translational Medicine, Semmelweis University, Budapest, Hungary
2. Department of Obstetrics and Gynecology, Semmelweis University, Budapest, Hungary
3. Centre for Translational Medicine, Semmelweis University, Budapest, Hungary
4. Institute of Pancreatic Diseases, Semmelweis University, Budapest, Hungary
5. Department of Urology, Semmelweis University, Budapest, Hungary
6. Institute for Translational Medicine, Medical School, University of Pécs, Pécs, Hungary.
7. Department of Biostatistics, University of Veterinary Medicine Budapest,Budapest, Hungary.
8. Institute of Bioanalysis, Medical School, University of Pécs, Pécs, Hungary

**TABLE OF CONTENT**

**Table S1.** PRISMA 2020 checklist

**Table S2.** Detailed exclusion and inclusion criteria of our selection

**Table S3.** GRADEpro assessment

**Table S4.** Eligibility criteria in each included article

**Supplementary Appendix S1.** Search key

**FIGURE OF CONTENT**

**Figure S1.** Risk of bias assessment for conversions to open surgery

**Figure S2.** Risk of bias assessment for estimated blood loss

**Figure S3.** Risk of bias assessment for intraoperative complications

**Figure S4.** Risk of bias assessment for length of stay in hospital

**Figure S5.** Risk of bias assessment for operating room time

**Figure S6.** Risk of bias assessment for operating time

**Figure S7.** Risk of bias assessment for postoperative complications

**Figure S8.** Risk of bias assessment for rehospitalizations

**Figure S9.** Risk of bias assessment for randomized studies

**Figure S10.** Clavien-Dindo classification I.

**Figure S11.** Clavien-Dindo classification II.

**Figure S12.** Clavien-Dindo classification III.a

**Figure S13.** Conversion to open surgery

**Figure S14.** Rehospitalizations

**Figure S15.** Estimated blood loss

**Figure S16.** Length of stay in hospital

**Figure S17.** rASRM stage I.

**Figure S18.** rASRM stage II.

**Figure S19.** rASRM stage III.

**Figure S20.** rASRM stage IV.

**Supplementary Table S1.** PRISMA 2020 checklist

| **Section and topic** | **Item #** | **Checklist item** | **Location where item is reported (page)** |  |
| --- | --- | --- | --- | --- |
| **Title** | | | | |
| Title | 1 | Identify the report as a systematic review. | 1 |  |
| **Abstract** | | | | |
| Abstract | 2 | See the PRISMA 2020 for Abstracts checklist (table 2). | 4 |  |
| **Introduction** | | | | |
| Rationale | 3 | Describe the rationale for the review in the context of existing knowledge. | 5 |  |
| Objectives | 4 | Provide an explicit statement of the objective(s) or question(s) the review addresses. | 5 |  |
| **Methods** | | | | |
| Eligibility criteria | 5 | Specify the inclusion and exclusion criteria for the review and how studies were grouped for the syntheses. | 7 |  |
| Information sources | 6 | Specify all databases, registers, websites, organizations, reference lists and other sources searched or consulted to identify studies. Specify the date when each source was last searched or consulted. | 7 |  |
| Search strategy | 7 | Present the full search strategies for all databases, registers, and websites, including any filters and limits used. | 7 |  |
| Selection process | 8 | Specify the methods used to decide whether a study met the inclusion criteria of the review, including how many reviewers screened each record and each report retrieved, whether they worked independently, and if applicable, details of automation tools used in the process. | 7-8 |  |
| Data collection process | 9 | Specify the methods used to collect data from reports, including how many reviewers collected data from each report, whether they worked independently, any processes for obtaining or confirming data from study investigators, and if applicable, details of automation tools used in the process. | 7-8 |  |
| Data items | 10a | List and define all outcomes for which data were sought. Specify whether all results that were compatible with each outcome domain in each study were sought (e.g., for all measures, time points, analyses), and if not, the methods used to decide which results to collect. | 8 |  |
|  | 10b | List and define all other variables for which data were sought (e.g., participant and intervention characteristics, funding sources). Describe any assumptions made about any missing or unclear information. | 9 |  |
| Study risk of bias assessment | 11 | Specify the methods used to assess risk of bias in the included studies, including details of the tool(s) used, how many reviewers assessed each study and whether they worked independently, and if applicable, details of automation tools used in the process. | 8 |  |
| Effect measures | 12 | Specify for each outcome the effect measure(s) (e.g., risk ratio, mean difference) used in the synthesis or presentation of results. | 9 |  |
| Synthesis methods | 13a | Describe the processes used to decide which studies were eligible for each synthesis (e.g., tabulating the study intervention characteristics and comparing against the planned groups for each synthesis (item #5)). | 9 |  |
|  | 13b | Describe any methods required to prepare the data for presentation or synthesis, such as handling of missing summary statistics, or data conversions. | 9 |  |
|  | 13c | Describe any methods used to tabulate or visually display results of individual studies and syntheses. | 9 |  |
|  | 13d | Describe any methods used to synthesize results and provide a rationale for the choice(s). If meta-analysis was performed, describe the model(s), method(s) to identify the presence and extent of statistical heterogeneity, and software package(s) used. | 9 |  |
|  | 13e | Describe any methods used to explore possible causes of heterogeneity among study results (e.g. subgroup analysis, meta-regression). | 9 |  |
|  | 13f | Describe any sensitivity analyses conducted to assess robustness of the synthesized results. | 9 |  |
| Reporting bias assessment | 14 | Describe any methods used to assess risk of bias due to missing results in a synthesis (arising from reporting biases). | 8 |  |
| Certainty assessment | 15 | Describe any methods used to assess certainty (or confidence) in the body of evidence for an outcome. | 8 |  |
| **Results** | | | | |
| Study selection | 16a | Describe the results of the search and selection process, from the number of records identified in the search to the number of studies included in the review, ideally using a flow diagram (see fig 1). | 9 |  |
|  | 16b | Cite studies that might appear to meet the inclusion criteria, but which were excluded, and explain why they were excluded. | - |  |
| Study characteristics | 17 | Cite each included study and present its characteristics. | 7 |  |
| Risk of bias in studies | 18 | Present assessments of risk of bias for each included study. | 8 |  |
| Results of individual studies | 19 | For all outcomes, present, for each study: (a) summary statistics for each group (where appropriate) and (b) an effect estimate and its precision (e.g. confidence/credible interval), ideally using structured tables or plots. | 9 |  |
| Results of syntheses | 20a | For each synthesis, briefly summarize the characteristics and risk of bias among contributing studies. | 13 |  |
|  | 20b | Present results of all statistical syntheses conducted. If meta-analysis was done, present for each the summary estimate and its precision (e.g. confidence/credible interval) and measures of statistical heterogeneity. If comparing groups, describe the direction of the effect. | 13 |  |
|  | 20c | Present results of all investigations of possible causes of heterogeneity among study results. | 13 |  |
|  | 20d | Present results of all sensitivity analyses conducted to assess the robustness of the synthesized results. |  |  |
| Reporting biases | 21 | Present assessments of risk of bias due to missing results (arising from reporting biases) for each synthesis assessed. | 13 |  |
| Certainty of evidence | 22 | Present assessments of certainty (or confidence) in the body of evidence for each outcome assessed. | 13 |  |
| **Discussion** | | | | |
| Discussion | 23a | Provide a general interpretation of the results in the context of other evidence. | 13 |  |
|  | 23b | Discuss any limitations of the evidence included in the review. | 18 |  |
|  | 23c | Discuss any limitations of the review processes used. | 18 |  |
|  | 23d | Discuss implications of the results for practice, policy, and future research. | 18 |  |
| **Other information** | | | | |
| Registration and protocol | 24a | Provide registration information for the review, including register name and registration number, or state that the review was not registered. | 6 |  |
|  | 24b | Indicate where the review protocol can be accessed, or state that a protocol was not prepared. | 6 |  |
|  | 24c | Describe and explain any amendments to information provided at registration or in the protocol. | 6 |  |
| Support | 25 | Describe sources of financial or non-financial support for the review, and the role of the funders or sponsors in the review. | 3 |  |
| Competing interests | 26 | Declare any competing interests of review authors. | 3 |  |
| Availability of data, code, and other materials | 27 | Report which of the following are publicly available and where they can be found: template data collection forms; data extracted from included studies; data used for all analyses; analytic code; any other materials used in the review. | 7 |  |

**Table S2.** Detailed exclusion and inclusion criteria of our selection

| Inclusion criteria | Exclusion criteria |
| --- | --- |
| -randomized controlled studies, observational studies  -pre-menopausal women, who underwent conventional or robot-assisted laparoscopy for endometriosis.  -the indication for surgery is endometriosis, but a preliminary histological diagnosis is not necessary, it is also sufficient if endometriosis is suspected based on clinical signs or symptoms.  -in robot-assisted cases, the type of robot does not matter.  - the degree of severity of endometriosis does not matter  -within an article, definitions for the outcomes of interest must be identical for the two groups  - conventional multi/single-port laparoscopy should be mentioned with robot-assisted multi/single-port in one study | -case reports, case series, conference abstracts, letters, protocol trials, reviews  -articles investigated just diagnostic laparoscopic intervention |

**Table S3.** GRADEpro assessment

**Author(s):**

**Question:** Robot-assisted laparoscopy compared to Conventional laparoscopy for premenopausal women who underwent surgery for endometriosis

**Setting:**

**Bibliography:**

| **Certainty assessment** | | | | | | | **№ of patients** | | **Effect** | | **Certainty** | **Importance** |
| --- | --- | --- | --- | --- | --- | --- | --- | --- | --- | --- | --- | --- |
| **№ of studies** | **Study design** | **Risk of bias** | **Inconsistency** | **Indirectness** | **Imprecision** | **Other considerations** | **Robot-assisted laparoscopy** | **Conventional laparoscopy** | **Relative (95% CI)** | **Absolute (95% CI)** |  |  |
| **Intraoperative complications (assessed with: event number)** | | | | | | | | | | | | |
| 11 | observational studies | serious | not serious | not serious | not serious | none | 11/904 (1.2%) | 12/903 (1.3%) | **OR 1.07** (0.43 to 2.63) | **1 more per 1 000** (from 8 fewer to 21 more) | ⨁⨁⨁◯ Moderate | CRITICAL |
| **Coversion (assessed with: event number)** | | | | | | | | | | | | |
| 10 | observational studies | serious | not serious | not serious | not serious | none | 6/807 (0.7%) | 6/606 (1.0%) | **OR 1.34** (0.76 to 2.37) | **3 more per 1 000** (from 2 fewer to 13 more) | ⨁⨁⨁◯ Moderate | IMPORTANT |
| **Length of stay in hospital (assessed with: days; Scale from: 1 to 14)** | | | | | | | | | | | | |
| 8 | observational studies | serious | not serious | not serious | not serious | none | 592 | 484 | - | MD **0.12 higher** (0.33 lower to 0.57 higher) | ⨁⨁⨁◯ Moderate | IMPORTANT |
| **Operative time (assessed with: minutes; Scale from: 0 to 400)** | | | | | | | | | | | | |
| 12 | observational studies | serious | not serious | not serious | not serious | none | 994 | 933 | - | MD **28.09 higher** (11.59 higher to 44.59 higher) | ⨁⨁⨁◯ Moderate | IMPORTANT |
| **Estimated blood loss (assessed with: ml; Scale from: 0 to 2500)** | | | | | | | | | | | | |
| 11 | observational studies | serious | not serious | not serious | not serious | none | 954 | 879 | - | MD **16.73 higher** (4.18 lower to 37.63 higher) | ⨁⨁⨁◯ Moderate | IMPORTANT |
| **Postoperative complications (assessed with: event number)** | | | | | | | | | | | | |
| 11 | observational studies | serious | not serious | not serious | not serious | none | 72/904 (8.0%) | 91/903 (10.1%) | **OR 1.30** (0.73 to 2.32) | **26 more per 1 000** (from 25 fewer to 106 more) | ⨁⨁⨁◯ Moderate | CRITICAL |
| **Rehospotalisation (follow-up: median 1 months; assessed with: event number)** | | | | | | | | | | | | |
| 3 | observational studies | serious | not serious | not serious | not serious | none | 8/119 (6.7%) | 9/124 (7.3%) | **OR 0.95** (0.13 to 6.75) | **3 fewer per 1 000** (from 63 fewer to 273 more) | ⨁⨁⨁◯ Moderate | IMPORTANT |

**CI:** confidence interval; **MD:** mean difference; **OR:** odds ratio

**Table S4.** Eligibility criteria in each included article

| **Author (year)** | **Inclusion criteria (“verbatim”)** | **Exclusion criteria (“verbatim”)** |
| --- | --- | --- |
| Ferrier (2022)(1) | Symptomatic patients scheduled for colorectal surgery, >18 years of age, after failure of medical treatment, or with infertility. | Patients with DE without confirmation of colorectal  endometriosis or refusing bowel surgery were excluded from the  study as were patients with prior colorectal surgery. |
| Raimondo (2021)(2) | From September 2018 to September 2019, we enrolled symptomatic patients with clinical and sonographic diagnosis of RSE. All patients enrolled were aged over 18 years and were asked  to provide written informed consent. | Postmenopausal status, suspected gynecological malignancy, and medical diseases precluding a minimally invasive approach. |
| Hiltunen (2021)(3) | Who had been operated  on for endometriosis-related pain between  January 2014 and December 2017 in  Kuopio University Hospital. | Bowel resection were excluded |
| Lee (2020)(4) | Women who underwent ovarian cystectomy for endometrioma by robotic assisted single port (n = 40) or laparoscopic single port (n = 54) surgery between May 2013 and November 2018 at the Department of Obstetrics and Gynecology, Kyungpook National University, Daegu, Korea. | None. |
| Le Gac (2020)(5) | The inclusion criteria were patients over 18 years-old, after failure of medical treatment or with infertility; with a preoperative diagnosis of DE with colorectal involvement confirmed by transvaginal ultrasonography, magnetic resonance imaging (MRI), and rectal echo endoscopy (REE) using previously published criteria, schedule for colorectal resection. | Patients with DE without proved colorectal endometriosis or requiring an Assisted Reproductive Technique (ART) and refusing bowel surgery were excluded of the study. Patients with prior colorectal surgery were also excluded. |
| Moon (2018)(6) | We included all the consecutive endometriosis cases that underwent SPL or RSS, therefore, the baseline clinical differences between the two groups (deep infiltrating endometriosis [DIE] and cyst size) were not intended. | None. |
| Soto (2017)(7) | Women were included if they were aged R18 years and were undergoing laparoscopic treatment of pain or infertility with presumed endometriosis as determined by the operating surgeon and/or ultrasound finding of endometrioma(s). | Exclusion criteria were suspected malignancy, medical illness precluding laparoscopy, inability to give informed consent, morbid obesity (body mass index >44 kg/m2 ), or need for concomitant bowel resection and/or ureteral reanastomosis. Patients preoperatively known to require bowel resection and/or ureteral reanastomosis were not included, given that these events impact operating time significantly and might not have been distributed equally between both arms. |
| Carpentier (2016)(8) | None. | None. |
| Nezhat (2015)(9) | Patients were included if they had undergone fertility-sparing treatment of endometriosis during the study period. | Patients were excluded if they were found to have stage 1 or 2 endometriosis or if they needed bladder, ureteral, or bowel resection (including disk excision) or hysterectomy, myomectomy, or thoracoscopy. |
| Magrina (2015)(10) | None. | None. |
| Nezhat (2014)(11) | Age 18 and above, general population through advertisements. The others were from couples with known female infertility factors and unknown semen quality, attending a fertility clinic aged ≥35 | Included all patients treated by either robotic-assisted or conventional laparoscopic surgery for stage III or IV endometriosis (American Society for Reproductive Medicine criteria) between July 2009 and October 2012 by 1 surgeon experienced in both techniques. |
| Dulemba (2013)(12) | All surgeries were performed at North Texas Hospital, a small private hospital in Denton, Texas. A single gynecological surgeon (JFD), specializing in the diagnosis and treatment of chronic pelvic pain and dysfunctional uterine bleeding due to endometriosis, completed all procedures described in this study. | None. |
| Nezhat (2010)(13) | None. | None. |

**Supplementary Appendix S1.** Search key

robot AND laparoscop* AND endometrio*

**Figure S1.** Risk of bias assessment at study and at domain level for conversions to open surgery


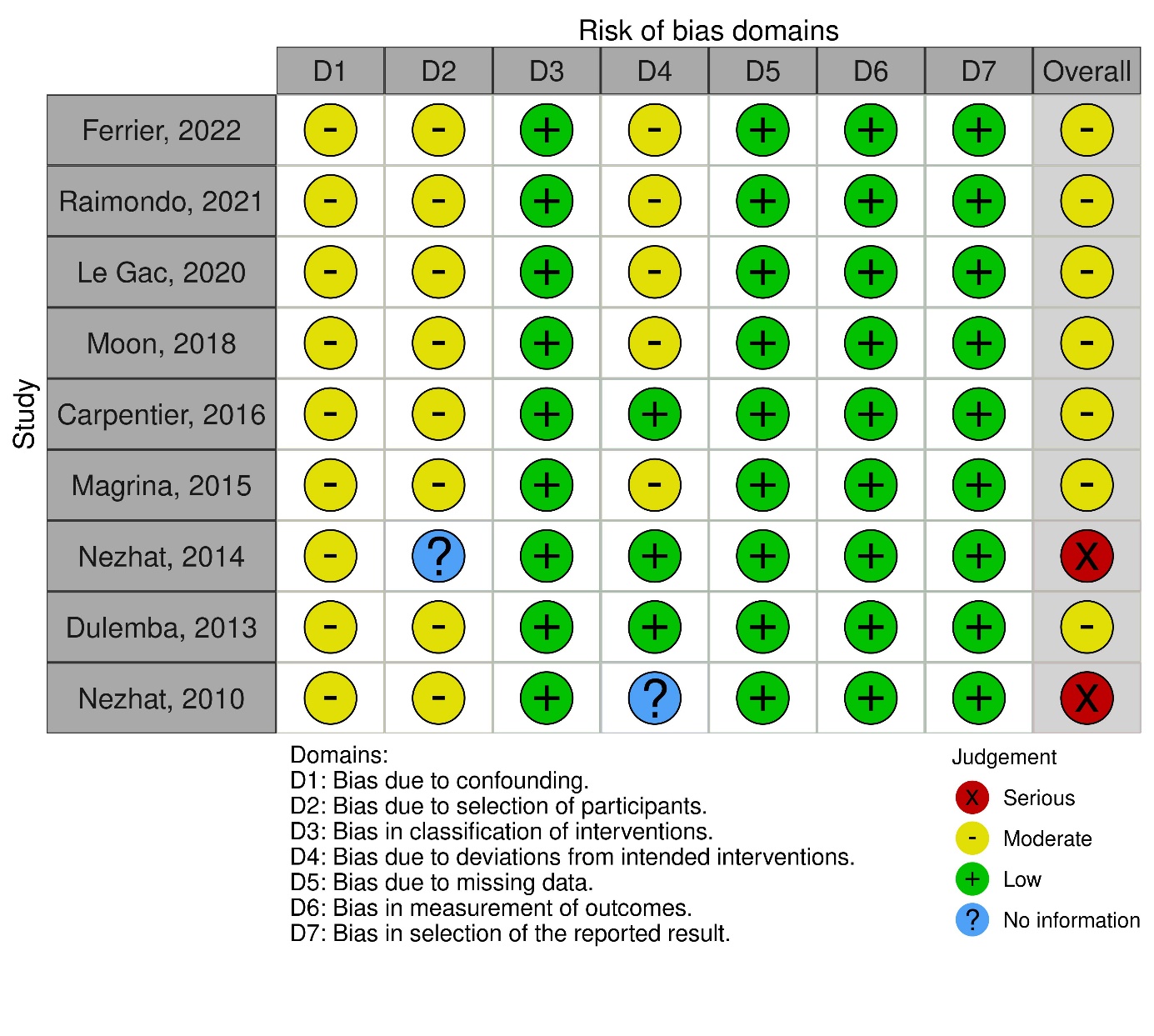

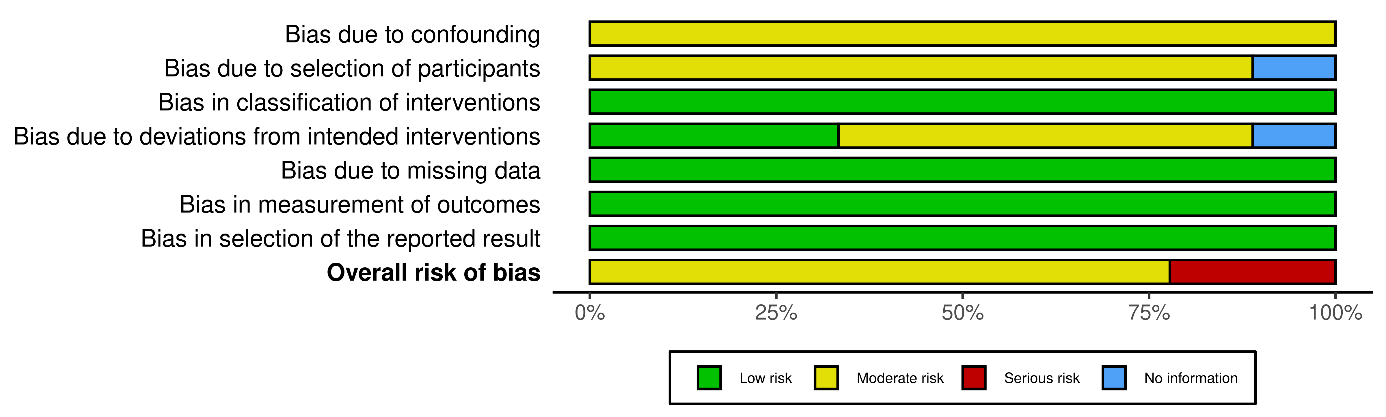


**Figure S2.** Risk of bias assessment at study and at domain level for estimated blood loss


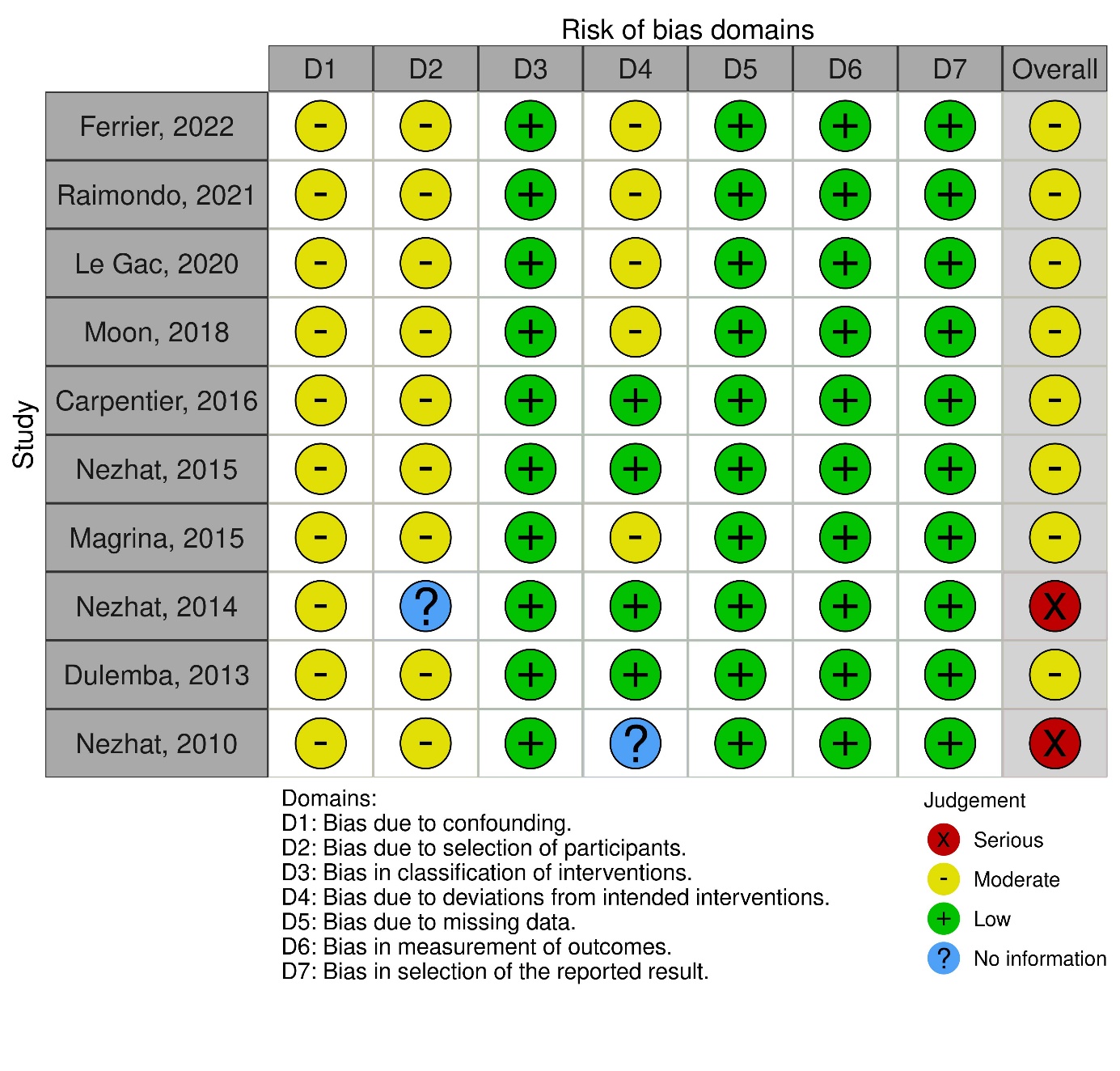


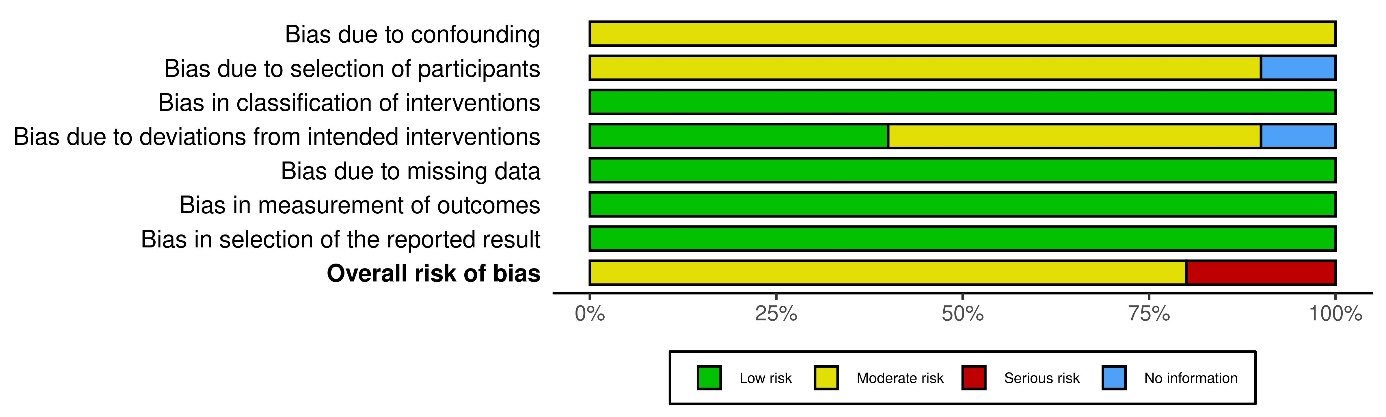


**Figure S3.** Risk of bias assessment at study and at domain level for intraoperative complications


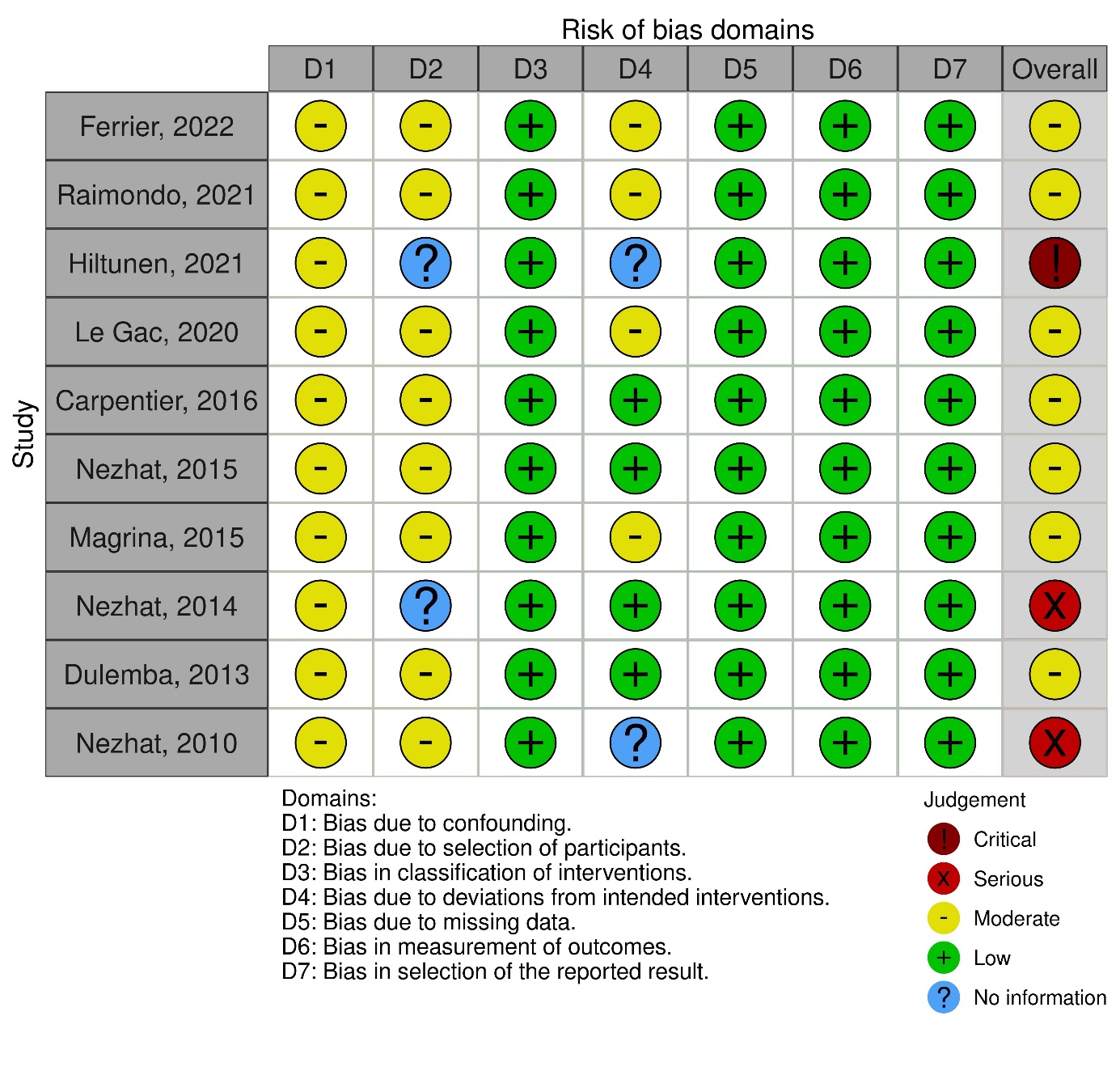


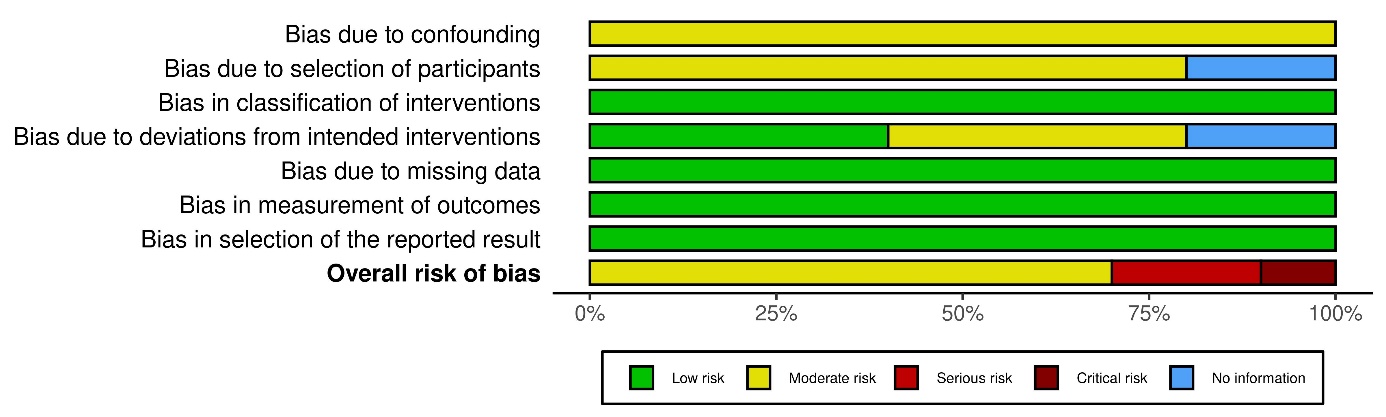


**Figure S4.** Risk of bias assessment at study and at domain level for length of stay in hospital


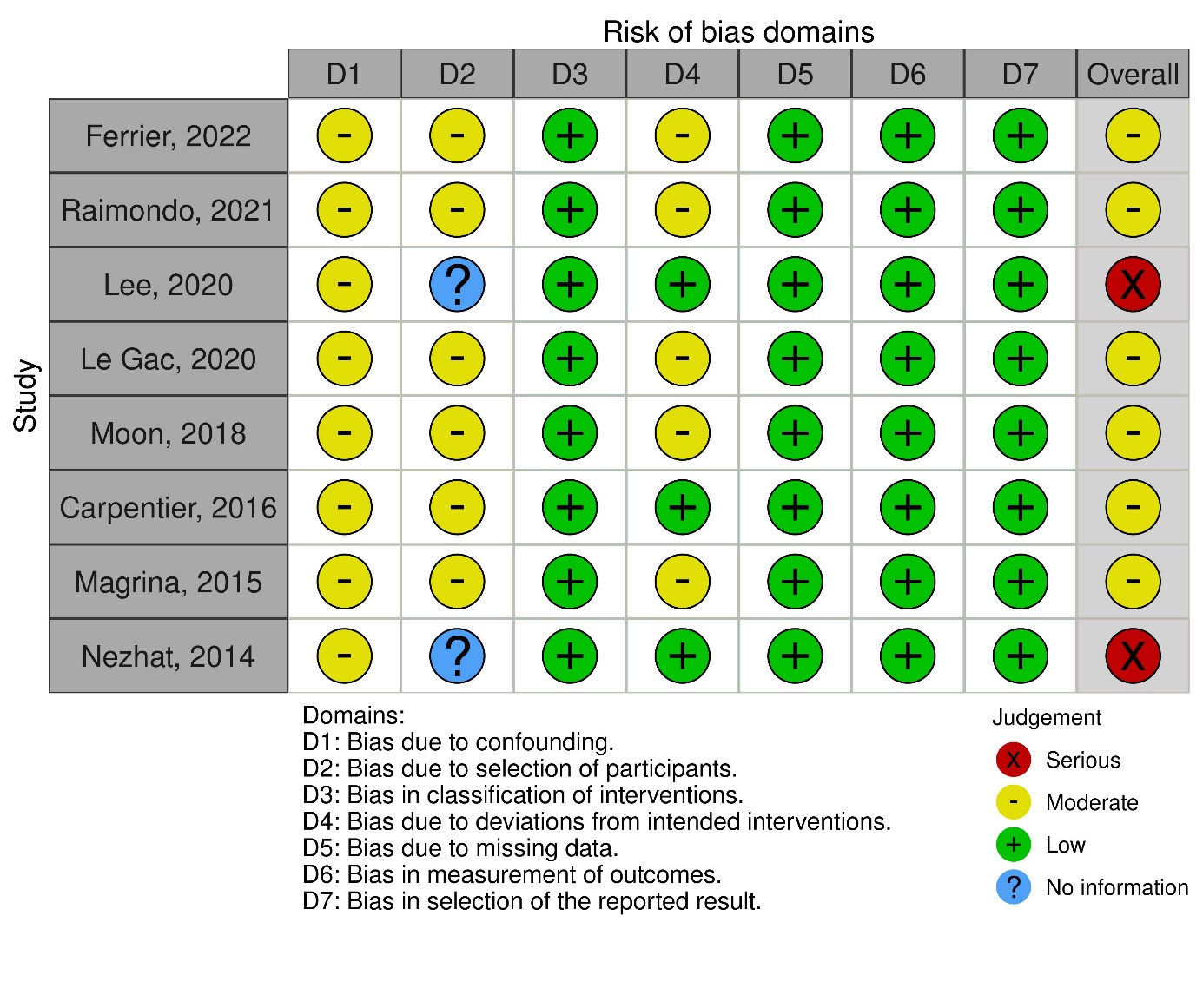


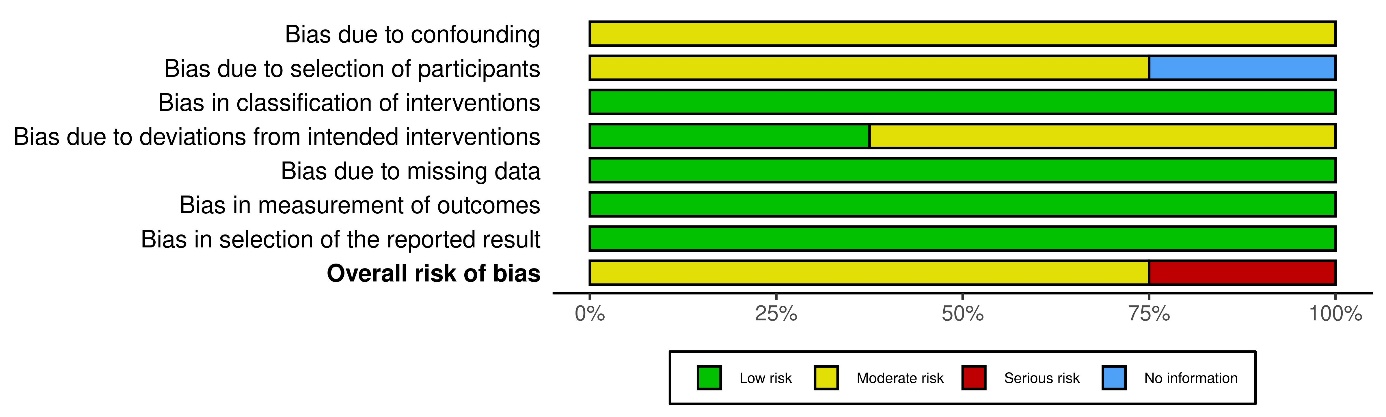


**Figure S5.** Risk of bias assessment at study and at domain level for operating room time


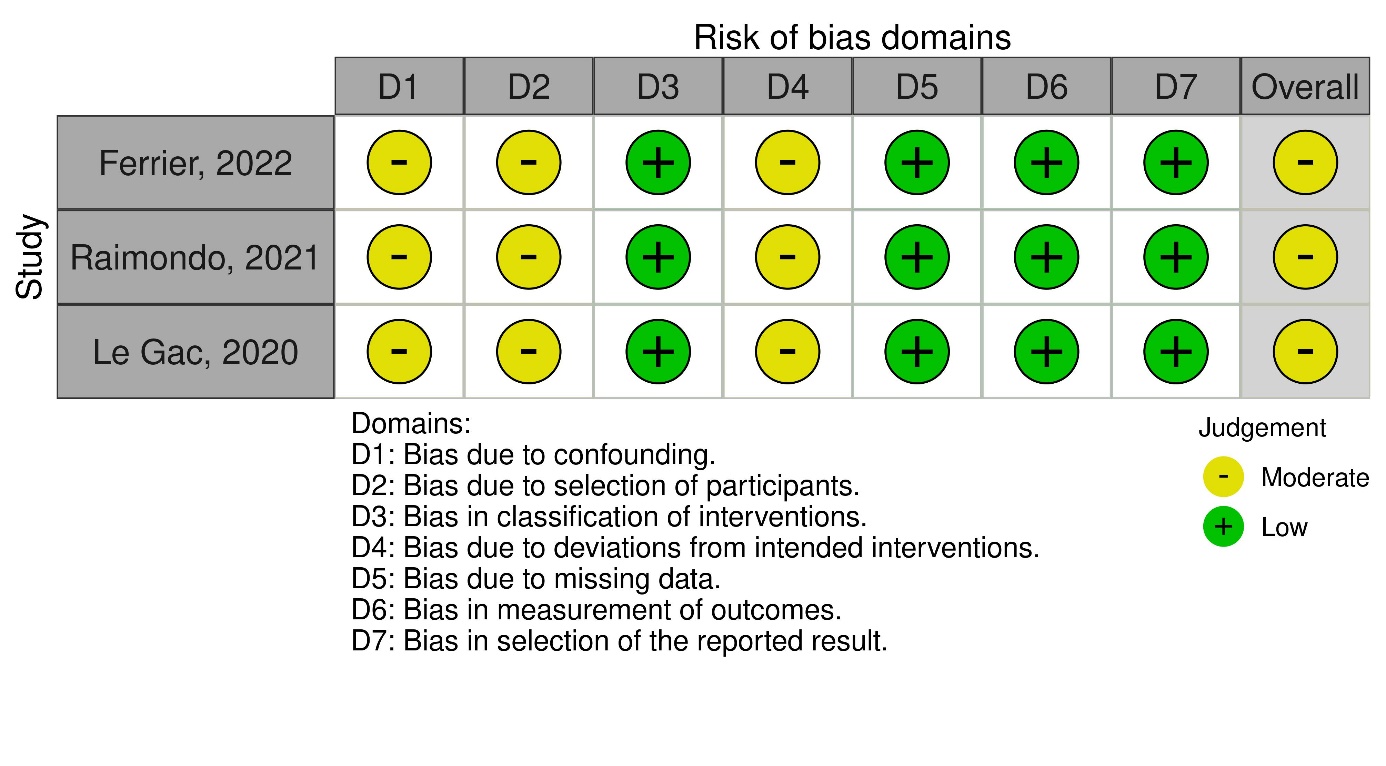


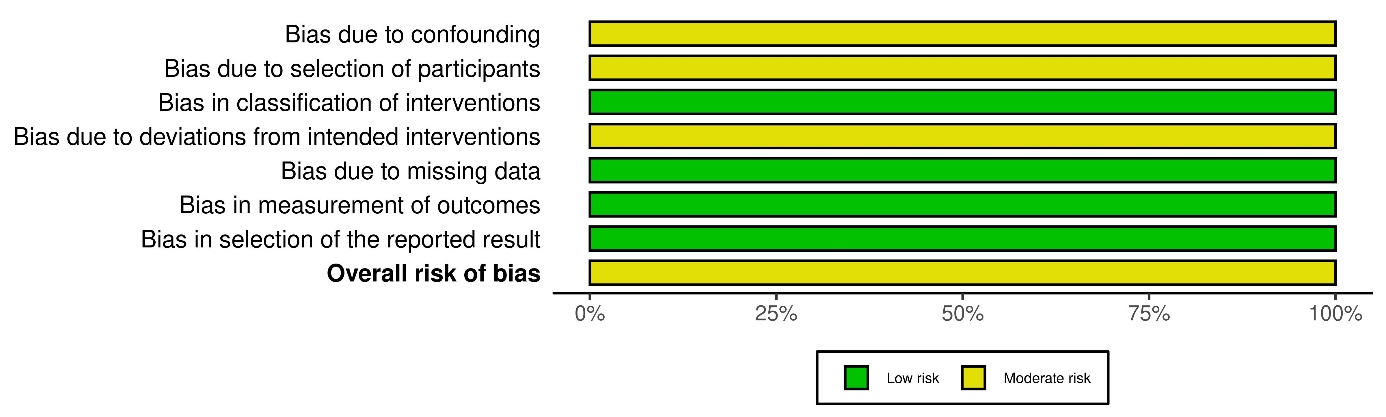


**Figure S6.** Risk of bias assessment at study and at domain level for operating time


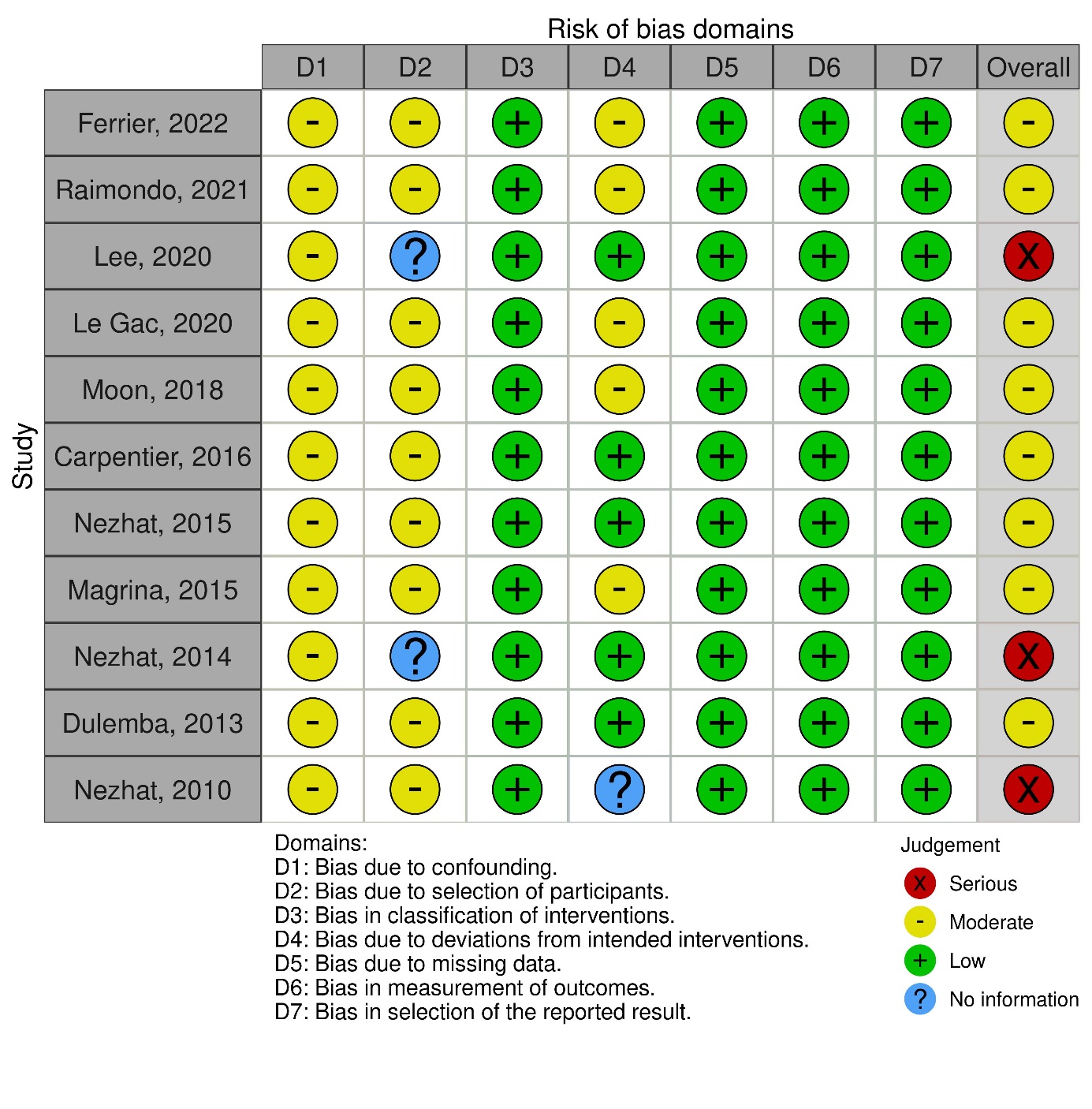


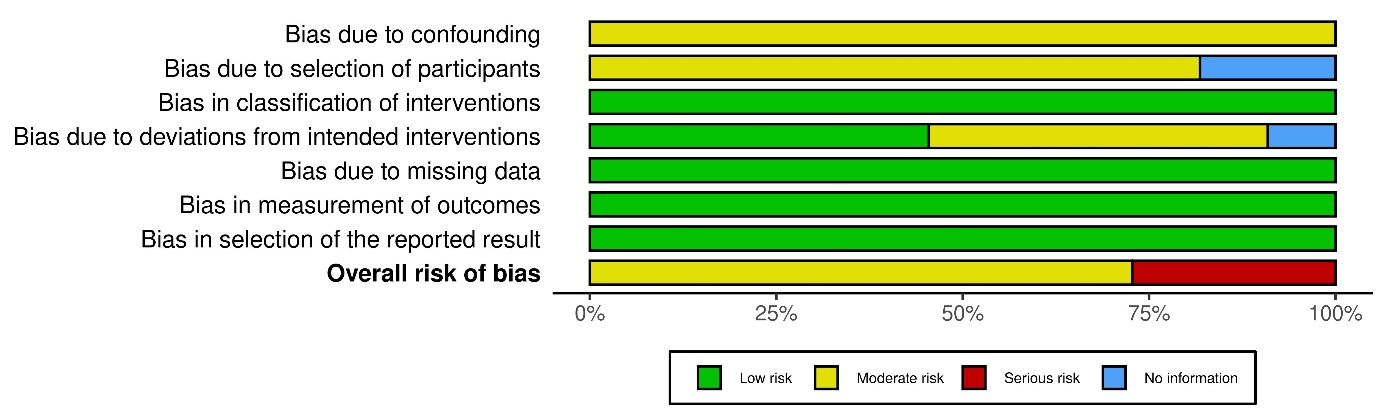


**Figure S7.** Risk of bias assessment at study and at domain level for postoperative complications


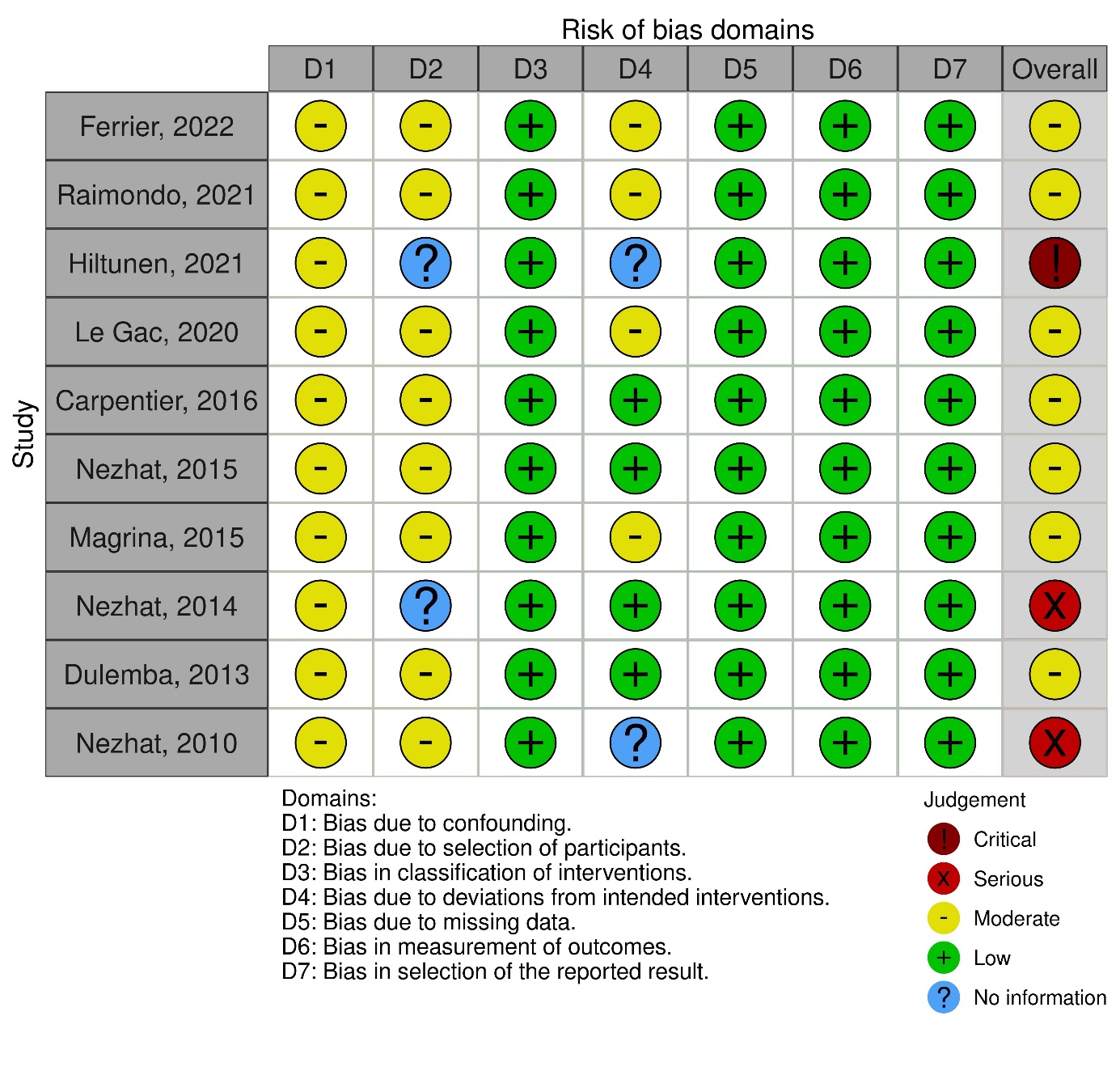


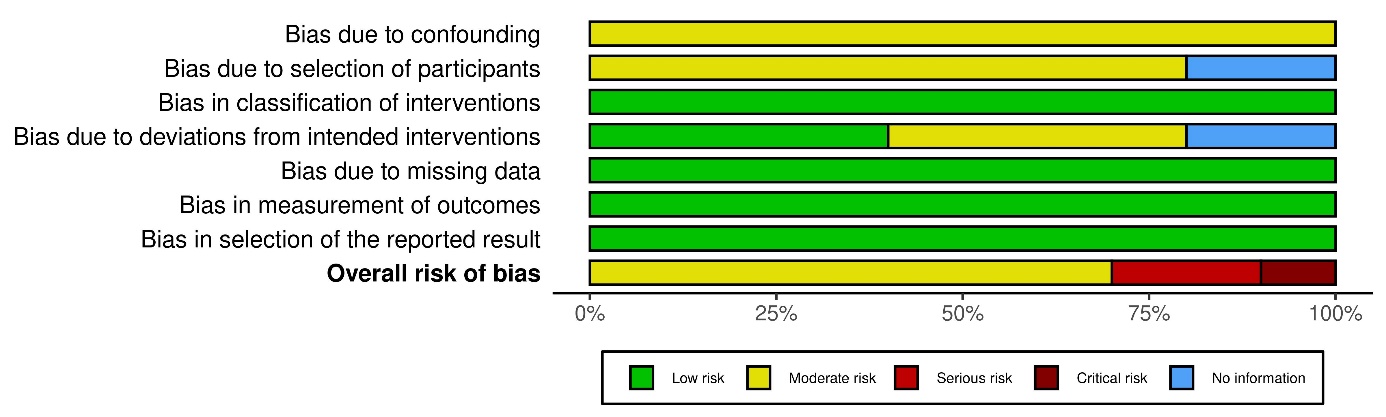


**Figure S8.** Risk of bias assessment at study and at domain level for rehospitalizations


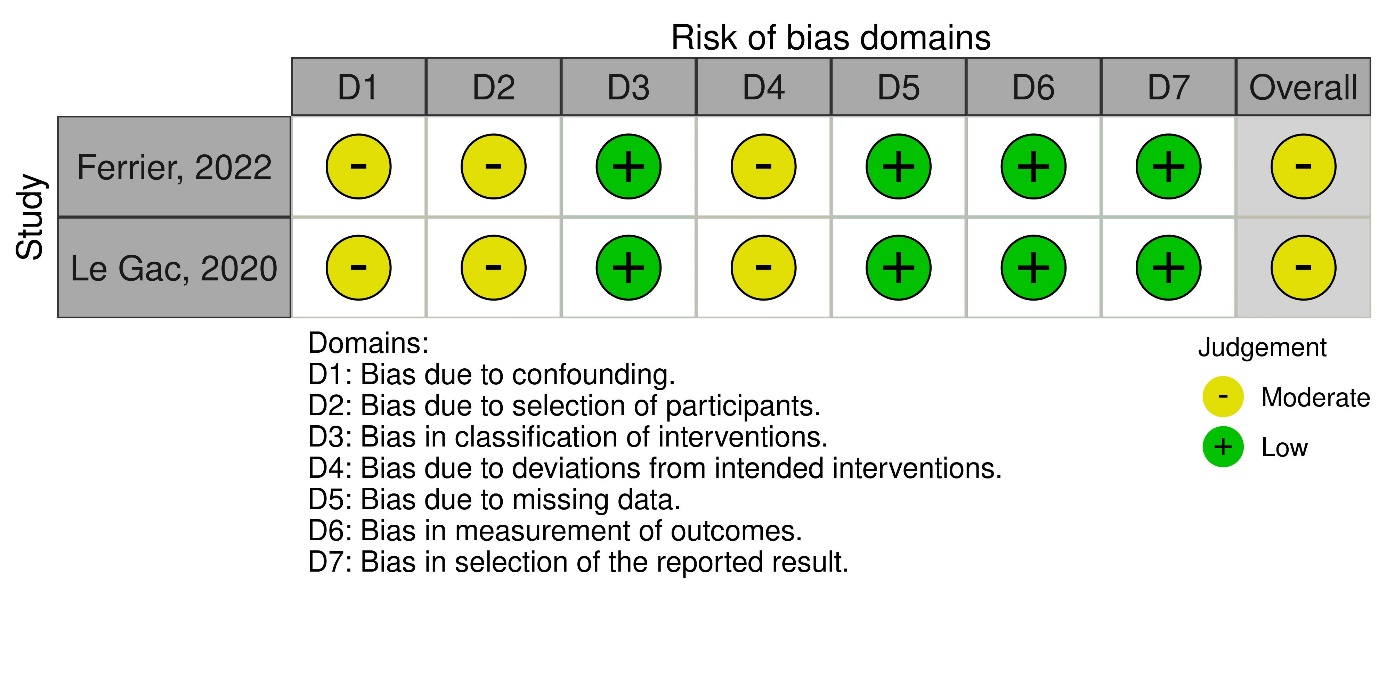


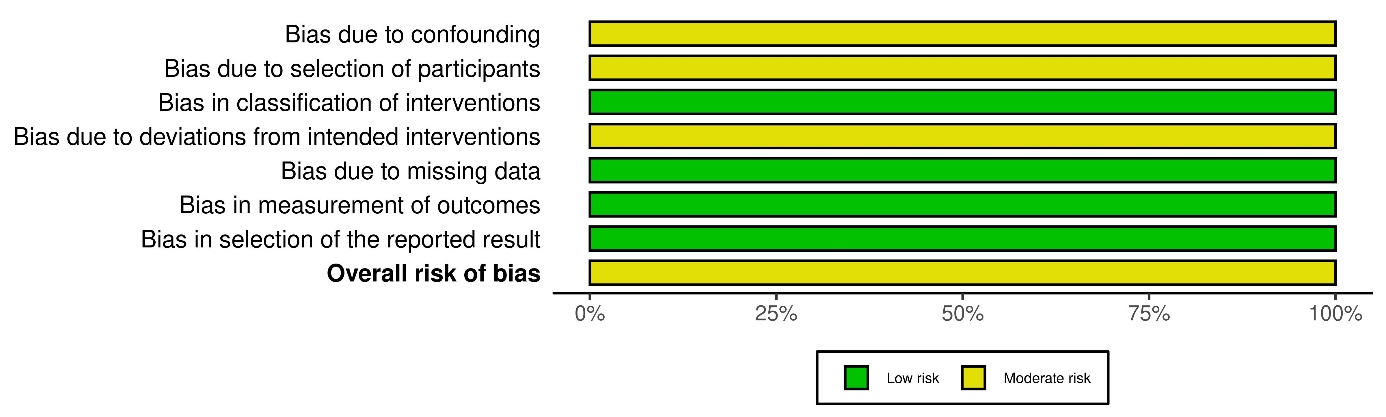


**Figure S9.** Risk of bias assessment for randomized study


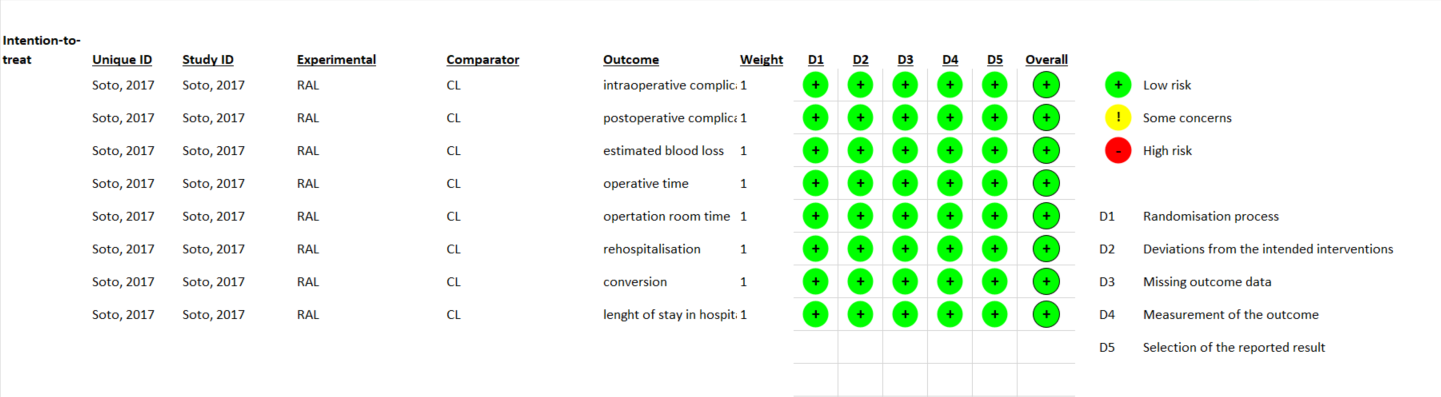


**Figure S10.** Clavien-Dindo classification I.


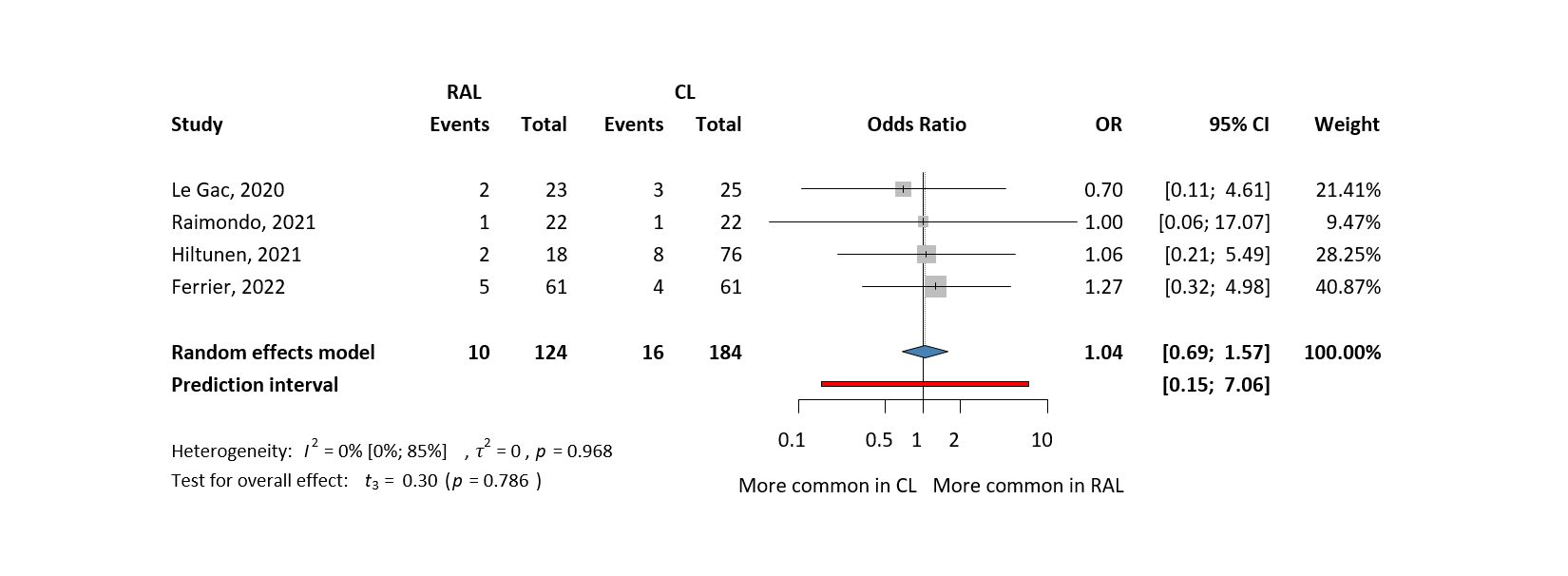


**Figure S11.** Clavien-Dindo classification II.


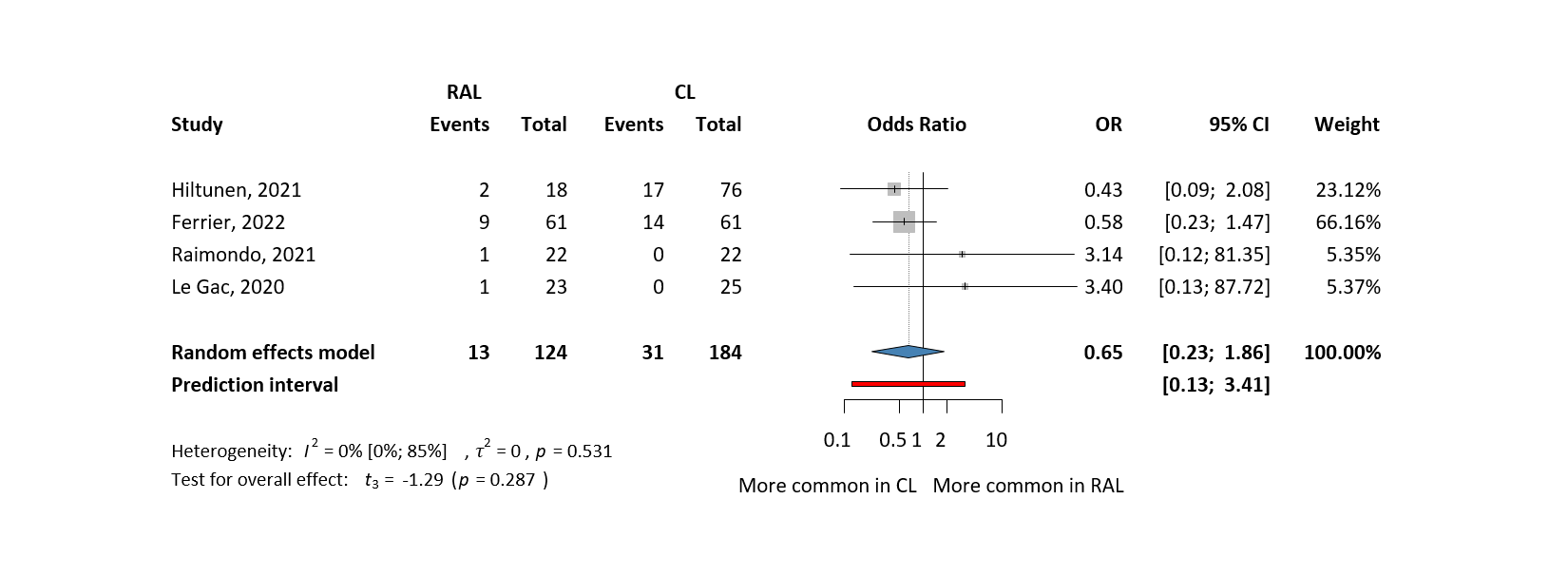


**Figure S12.** Clavien-Dindo classification III.a


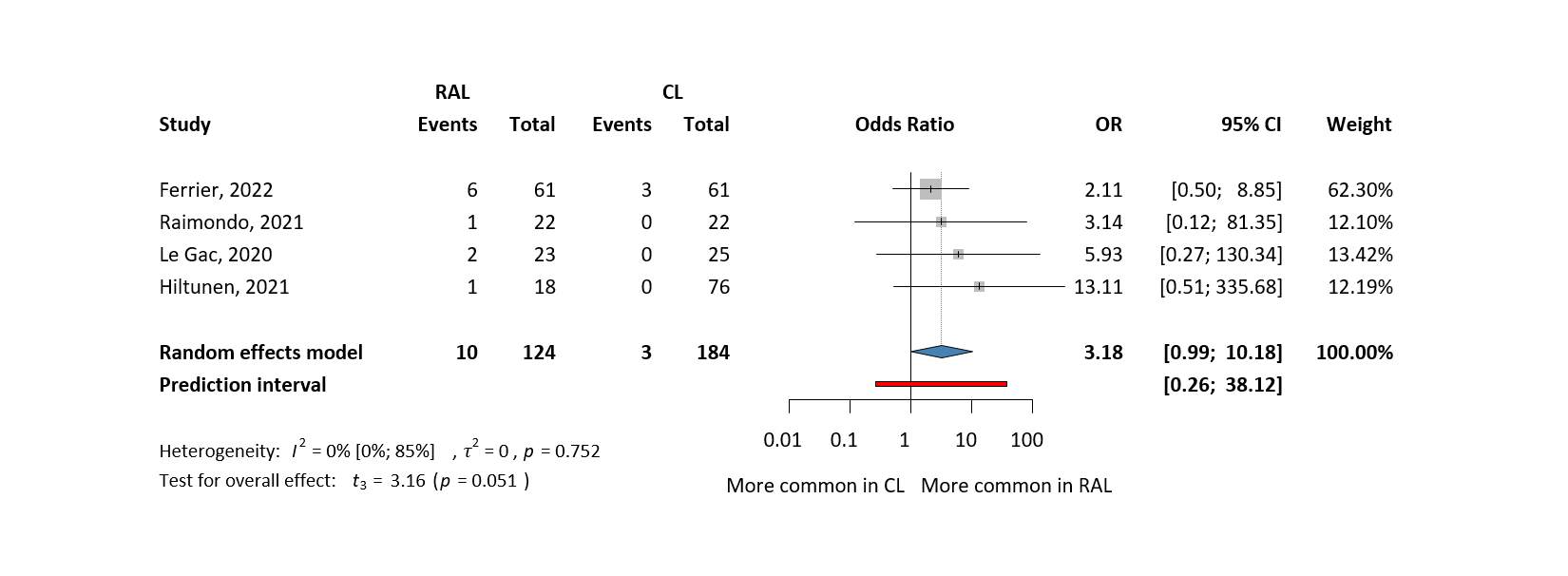


**Figure S13.** The comparison of RAL and CL in terms of odds ratio of conversions to open surgery (event numbers)


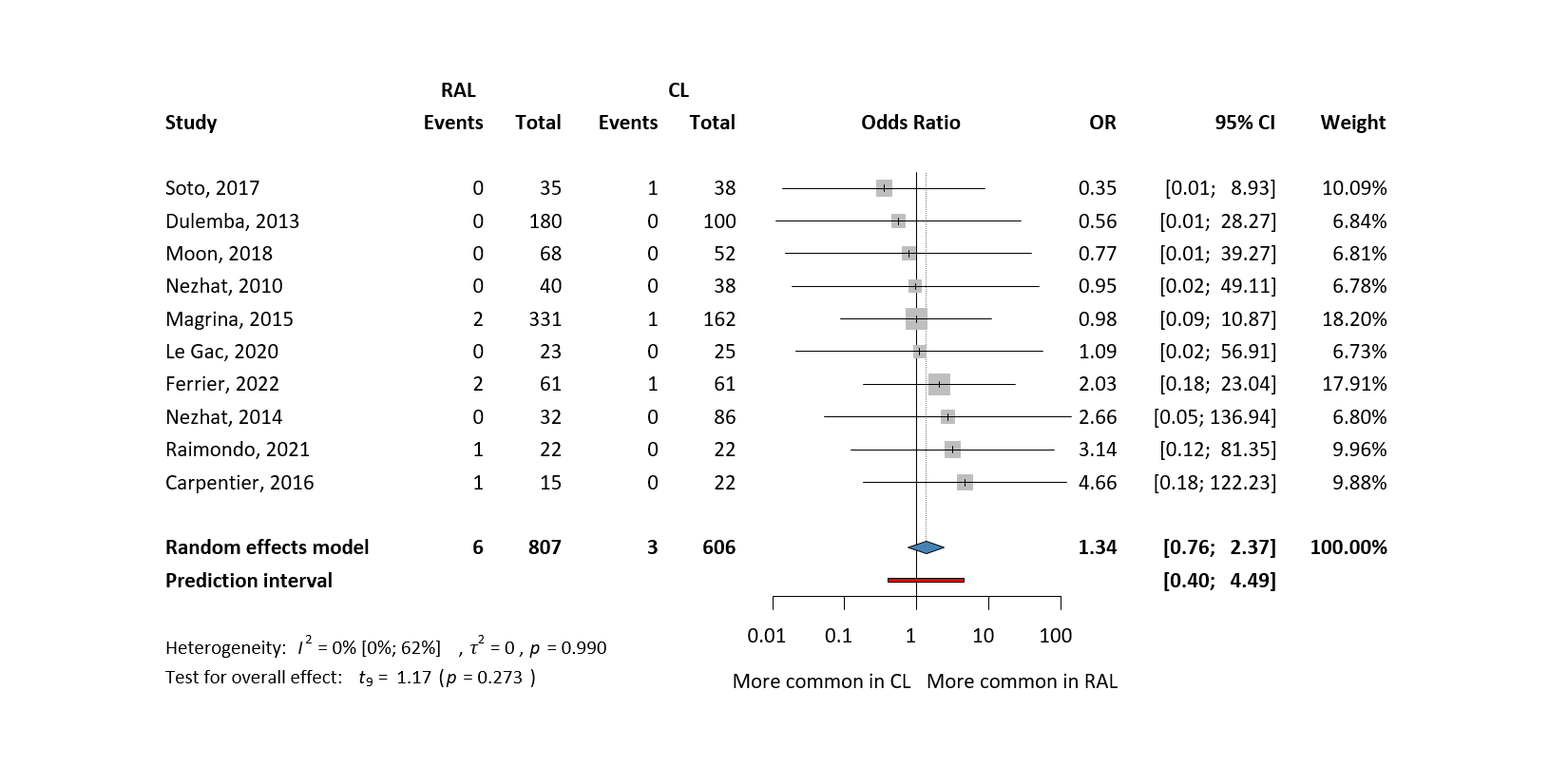


**Figure S14.** The comparison of RAL and CL in terms of odds ratio of rehospitalizations (event numbers)


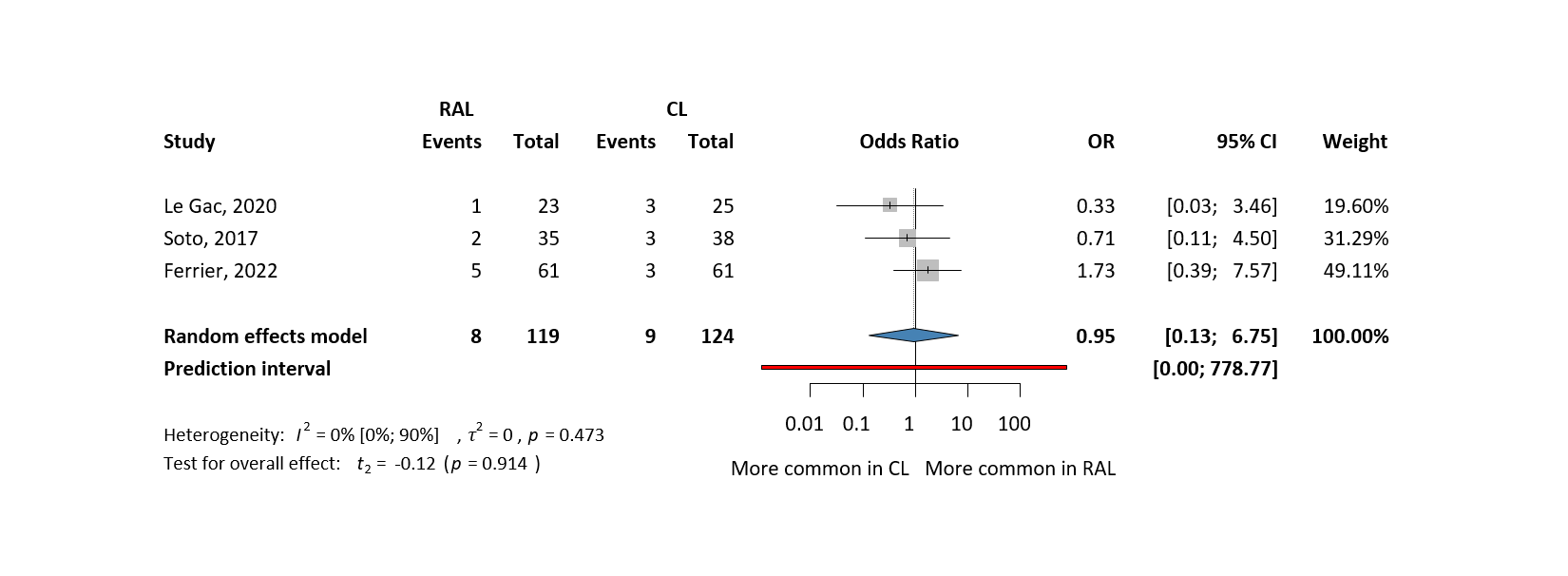


**Figure S15.** The comparison of RAL and CL in terms of mean difference of estimated blood loss (millilitres)


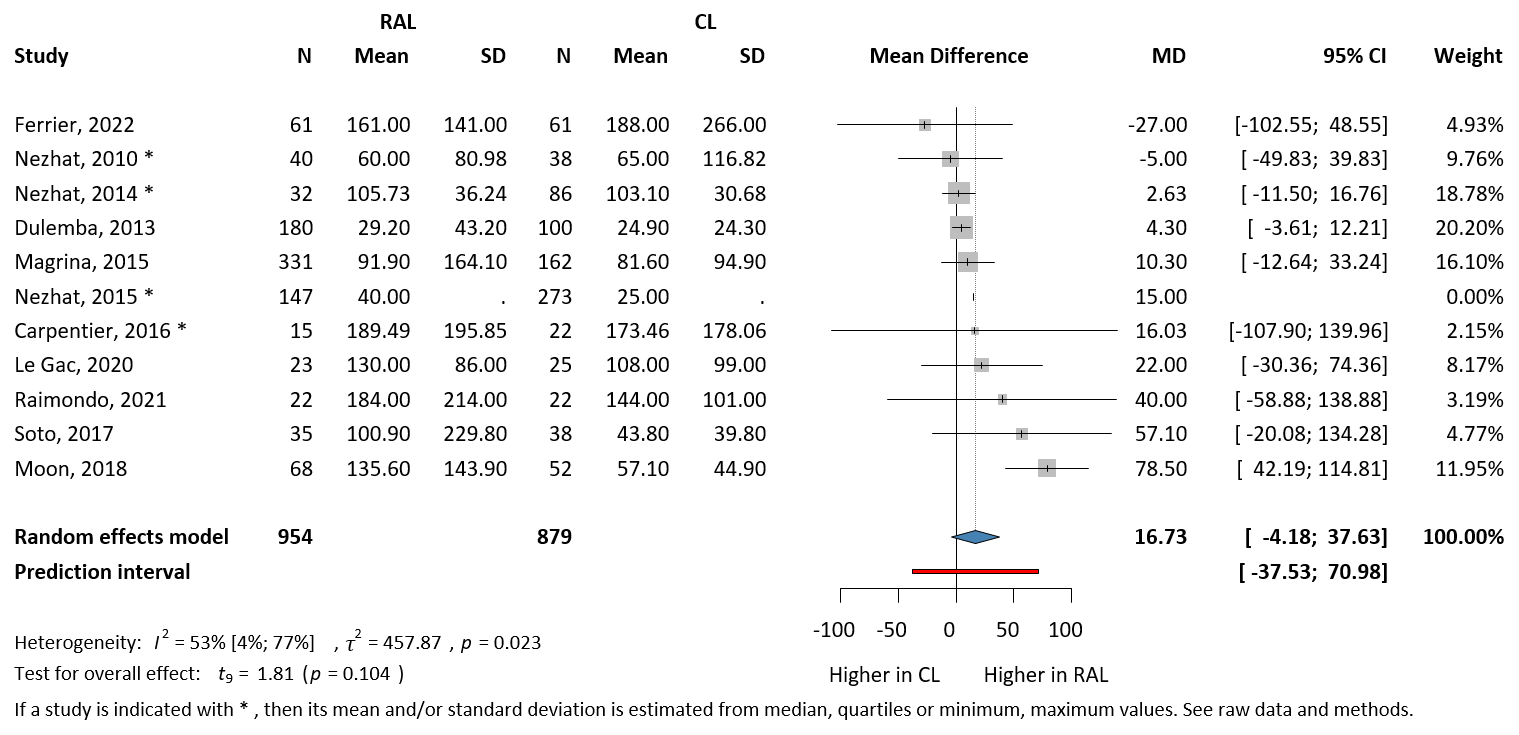


**Figure S16.** The comparison of RAL and CL in terms of mean difference of length of hospital stay (days)


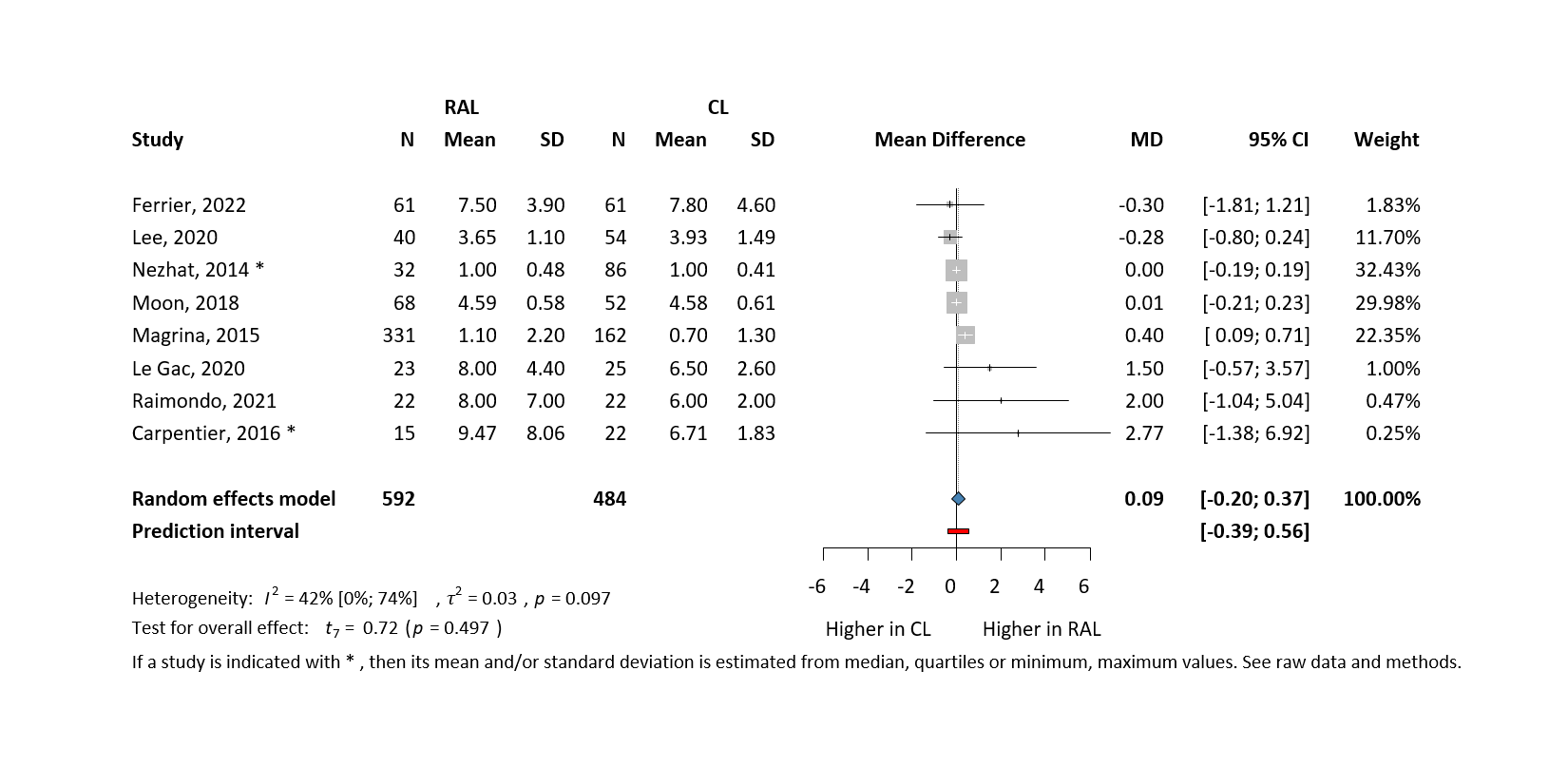


**Figure S17.** Distribution of patients according to rASRM stage I. between RAL and CL.


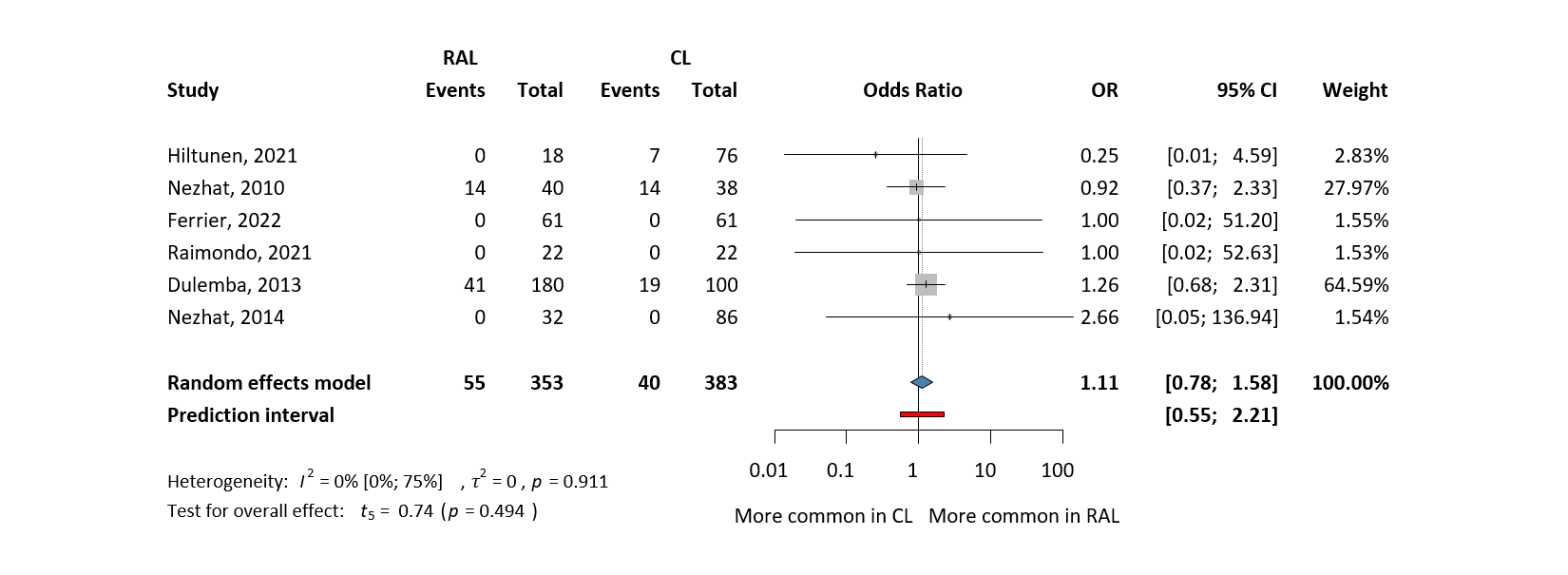


**Figure S18.** Distribution of patients according to rASRM stage II. between RAL and CL.


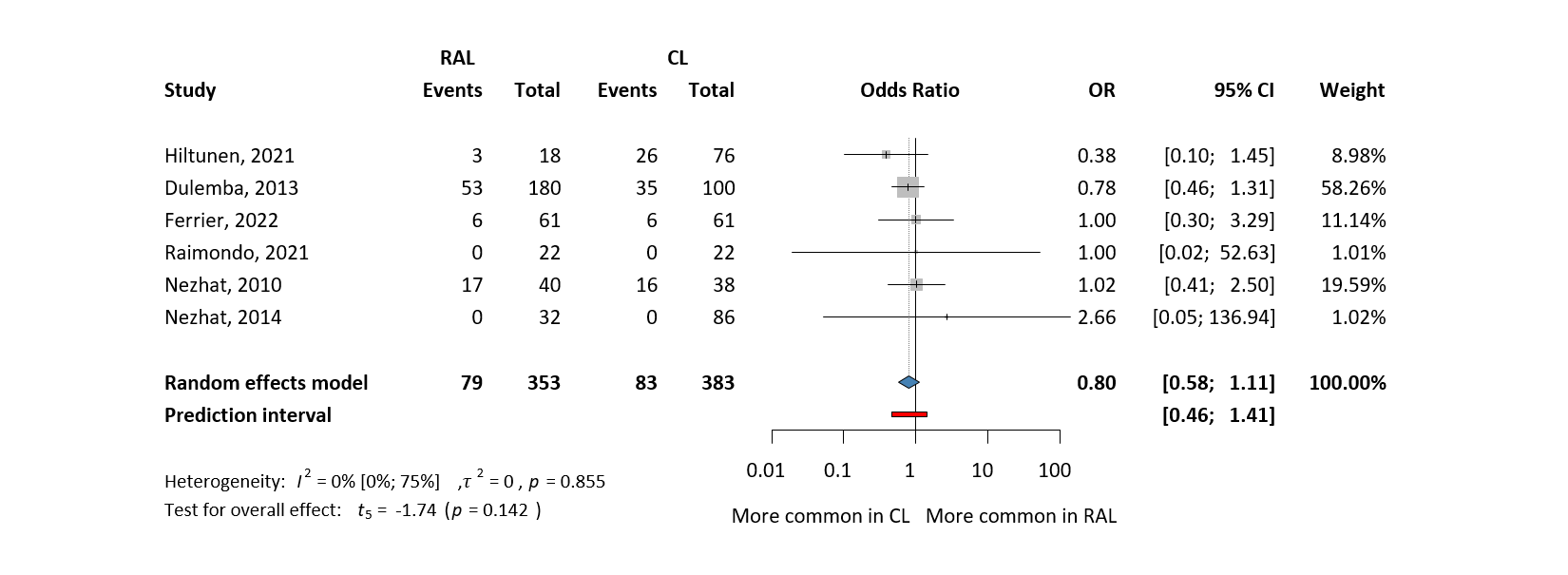


**Figure S19.** Distribution of patients according to rASRM stage III. between RAL and CL.


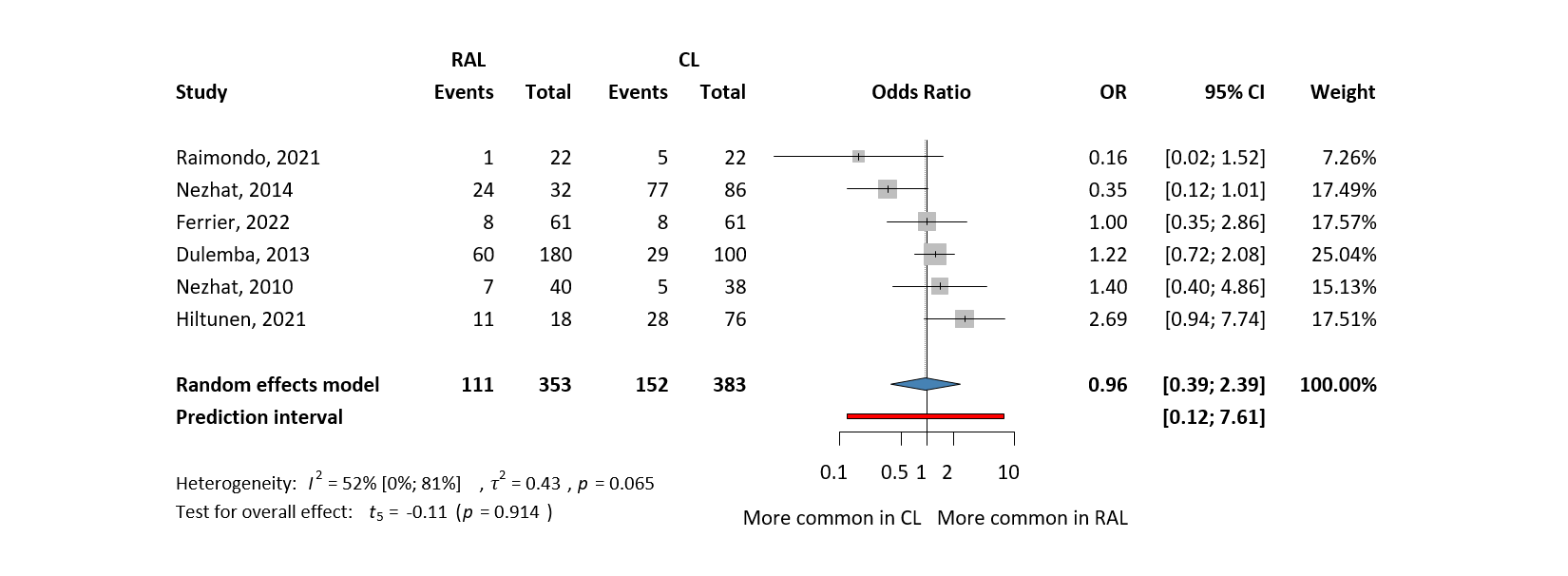


**Figure S20.** Distribution of patients according to rASRM stage IV. between RAL and CL.


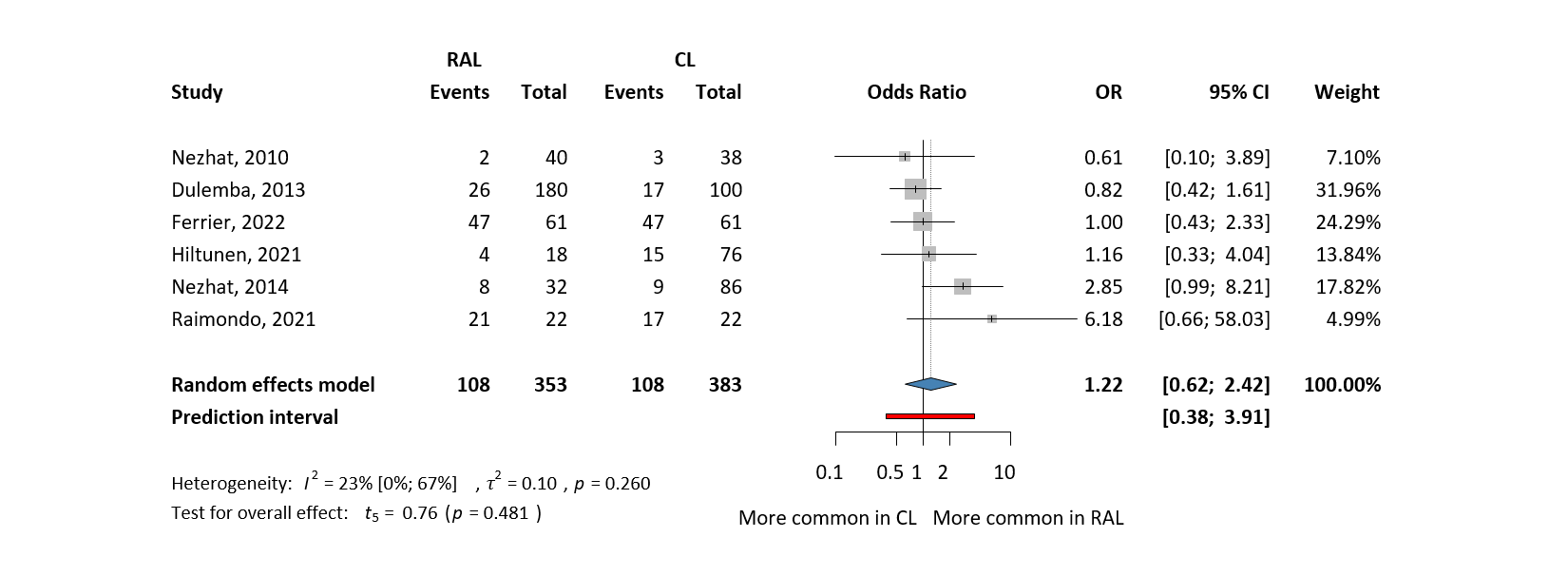

Supplement: Supplementary file 1 — Supplementary file1 (DOCX 4129 KB) [file 464_2023_10587_MOESM1_ESM.docx]
